# Prefusion structure, evasion and neutralization of HSV-1 glycoprotein B

**DOI:** 10.1038/s41564-025-02153-x

**Published:** 2025-10-31

**Authors:** Ryan S. Roark, Andrew J. Schaub, Wei Shi, Maple Wang, Fabiana A. Bahna, Jordan E. Becker, Andrea Biju, Sue Chong, Haijuan Du, Yicheng Guo, Hsiang Hong, Phinikoula S. Katsamba, Seetha M. Mannepalli, Adam S. Olia, Li Ou, Sarah K. Rubin, Yosef Sabo, Mehin Suleiman, Malcolm L. Wells, Baoshan Zhang, Cheng Cheng, Anum Glasgow, David D. Ho, Yaoxing Huang, Theodore C. Pierson, Reda Rawi, Tongqing Zhou, Lawrence Shapiro, Peter D. Kwong

**Affiliations:** 1 Aaron Diamond AIDS Research Center, Columbia University Vagelos College of Physicians and Surgeons, New York, NY USA; 2 Department of Biochemistry and Molecular Biophysics, Columbia University, New York, NY USA; 3 Zuckerman Mind Brain Behavior Institute, Columbia University, New York, NY USA; 4 Vaccine Research Center, National Institute of Allergy and Infectious Diseases, National Institutes of Health, Bethesda, MD USA

**Keywords:** Herpes virus, Antibodies, Cryoelectron microscopy, Vaccines, Viral immune evasion

## Abstract

Glycoprotein B (gB) refolds between prefusion and postfusion conformations to facilitate herpesvirus entry into host cells. However, the isolation of prefusion-specific neutralizing antibodies, effective against other viral entry machines, has been challenging. Here we describe stabilization of the prefusion gB ectodomain from herpes simplex virus 1 (HSV-1), determine ectodomain structures at 2.9- to 4.1-Å resolution using cryogenic electron microscopy (cryo-EM) and isolate a prefusion-specific gB-neutralizing antibody termed WS.HSV-1.24. Murine immunization with gB stabilized in the prefusion conformation induced high titres of antibodies binding to both prefusion and postfusion gB, but—most notably—without measurable serum neutralization. Accessibility analysis revealed iso-surface exposure, with accessible surfaces on prefusion HSV-1 gB also exposed on postfusion gB. Structural analysis suggested substantial plasticity, with regions that refolded between pre- and postfusion conformations relegated to domain interfaces with limited accessibility; indeed, WS.HSV-1.24 recognized a domain-interface refolding region to facilitate neutralization. We propose that prefusion HSV-1 gB evades neutralization by most antibodies through an iso-surface display that is coupled to structural plasticity.

## Abstract (web summary)

Cryo-EM structures of the stabilized prefusion conformation of the glycoprotein B ectodomain—the HSV-1 entry machine—identify a prefusion-specific neutralizing antibody and reveal how prefusion glycoprotein B may evade antibody-mediated neutralization.

## Main

Enveloped viruses use diverse mechanisms to evade immune responses, including hiding viral antigens behind a host-derived viral membrane^1,2^. However, in the cat-and-mouse game involving the immune system and virus, the fusion machines that facilitate the merging of viral and target-cell membranes are often themselves targeted by virus-neutralizing antibodies^3–6^, and these fusion machines may in turn possess mechanisms for immune evasion. Nonetheless, the dominant neutralizing response, as observed for class I fusion machines from human immunodeficiency virus type 1 (HIV-1), influenza A virus and respiratory syncytial virus (RSV), as well as for class II fusion machines such as that of the Dengue virus, arises from antibodies that recognize the prefusion conformation of the entry machines used by these viruses.

For HIV-1, antibodies that recognize the prefusion closed conformation of the trimeric envelope fusion machine dominate the neutralization of tier 2 neutralization-resistant isolates that have confounded vaccine development^4,7–9^. For influenza A virus, antibodies can inhibit virus replication through multiple mechanisms^10^, but antibodies that recognize the prefusion conformation of the haemagglutinin entry machine dominate the direct inhibition of entry, and vaccine developers are careful to maintain the prefusion conformation of haemagglutinin-based immunogens (for example, maintaining the pH at neutral and above)^5,11^. For RSV, antibodies against the prefusion conformation of the fusion glycoprotein have been found to be of high neutralization potency^3,12–14^; vaccines based on the stabilized prefusion conformation of the RSV fusion glycoprotein—but not those based on its postfusion conformation—have succeeded in clinical trials^15,16^. For Dengue virus, many of the most potent and broadly neutralizing antibodies recognize quaternary epitopes on the prefusion envelope dimer present on virions^6,17,18^, with dimers stabilized in the prefusion conformation eliciting higher neutralizing titres than their non-stabilized counterparts^19,20^.

Is prefusion susceptibility universal? In this Article, we analyse glycoprotein B (gB), the class III fusion machine^21,22^ underlying entry of herpes simplex virus 1 (HSV-1), a prototypical member of the herpesvirus family that is most commonly associated with recurrent oral or genital lesions^23,24^. Despite the high prevalence and global health burden of HSV-1 infections^25^, there are currently no effective vaccines or biotherapeutics with clinical approval^26^. The lack of modifications that fully lock HSV-1 gB in the metastable prefusion conformation has thus far prevented the evaluation of prefusion gB as a vaccine target and impeded the identification of prefusion gB-specific neutralizing antibodies. We used structure-based design to identify mutations encoding alterations capable of stabilizing the prefusion conformation of HSV-1 gB and determined both pre- and postfusion gB ectodomain structures. We immunized mice with gB stabilized in the prefusion conformation and assessed neutralization and binding. We analysed the structure of gB to delineate: (1) surfaces exposed in pre- and postfusion conformations; (2) domain movements and locations of refolding regions; (3) surface chemistries of solvent interaction; and (4) regions shielded by *N*-linked glycan. We further defined the neutralization mechanisms of the gB-directed antibodies WS.HSV-1.24 (a prefusion-specific gB-directed antibody isolated from mice immunized with prefusion gB) and D48 (a previously identified neutralizing human antibody^27^). Collectively, our findings revealed that HSV-1 gB is mostly impervious to prefusion neutralization, in contrast with the entry machines of HIV-1, influenza and RSV.

## Results

### Conformational stabilization and structure of prefusion gB

**Fig. 1:**
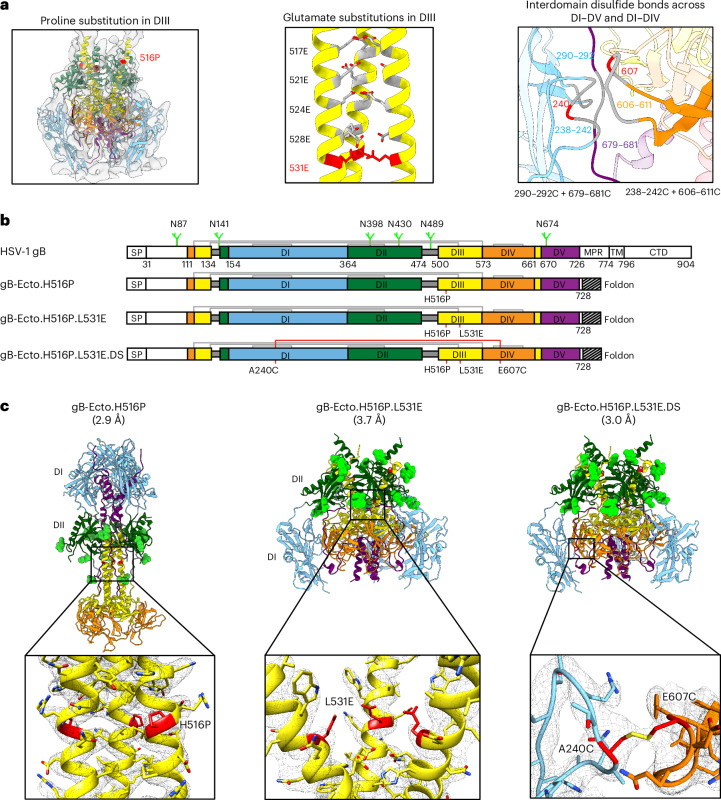
Conformational stabilization of prefusion HSV-1 gB. **a**, Strategies for stabilizing the prefusion conformation of the HSV-1 gB ectodomain include a helix-breaking proline that is known to partially destabilize the extended postfusion DIII helix (H516P; left), the charge destabilization of the postfusion DIII helical bundle through the introduction of glutamate substitutions (middle) and the locking of domains DI and DV or DI and DIV in their prefusion conformation with the introduction of artificial interprotomer disulfide bonds (right). Viable alterations utilized in this study are highlighted in red. The prefusion structure and 3D reconstruction density on the left are available from the Protein Data Bank (PDB 6Z9M) and Electron Microscopy Data Bank (EMDB 11123), respectively. For the structure on the right, DI, DIV and DV are shown in blue, orange and purple, respectively. **b**, Construct schematics for the full-length HSV-1 gB ectodomain and the HSV-1 gB ectodomain stabilized in the prefusion conformation. Naturally occurring disulfide bonds are depicted as thin grey lines between cysteine residues. The engineered disulfide bond is depicted with a red line. Alterations caused by point mutations are labelled. *N*-linked glycans are depicted in light green only in the full-length gB schematic for clarity. **c**, Structural models as determined by single-particle cryo-EM, with specific prefusion conformation-stabilizing alterations shown in red and magnified below. The 3D reconstruction density is shown as grey mesh. *N*-linked glycans are shown as spheres and coloured lime green.

**Extended Data Fig. 1:**
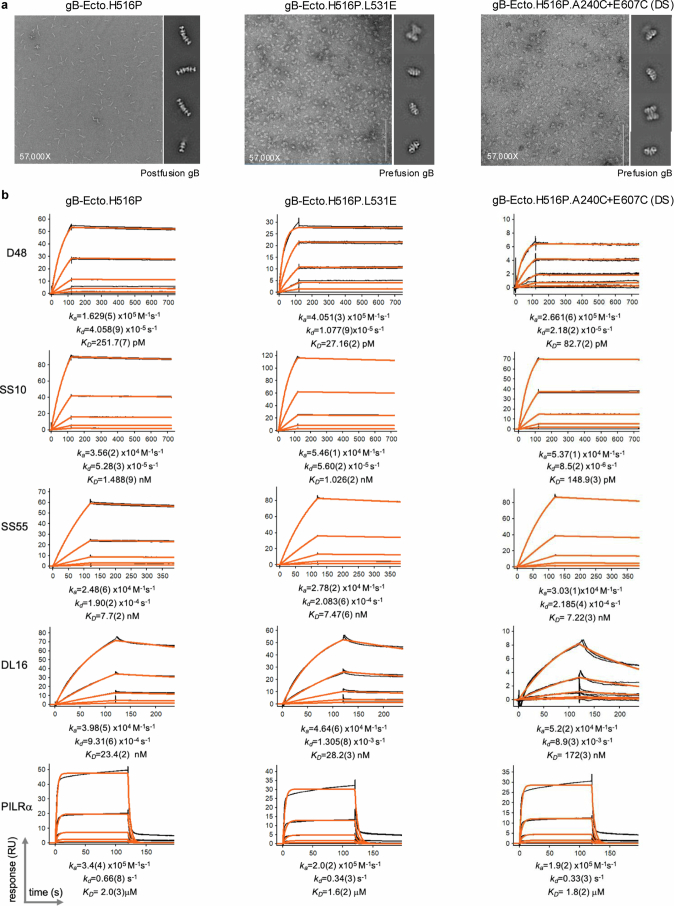
Conformational and antigenic assessment of HSV-1 gB ectodomain constructs. **a**, Representative negative stain EM micrographs and 2D class averages for gB constructs delineate prefusion and postfusion conformations. Approximately 100 micrographs were collected across two independent experiments for each gB construct. **b**, SPR kinetic binding assays with monoclonal antibodies and the HSV-1 gB co-receptor, PILRa, to compare the antigenicity of prefusion-stabilized and postfusion gB. Antibodies SS10, SS55 and DL16 have been previously reported to recognize discontinuous epitopes dependent on natively folded gB.

The HSV-1 gB ectodomain comprises five distinct domains, termed DI–DV, that have been structurally defined at sub-nanometre resolution (~9 Å) in the prefusion conformation^28^ and atomic-level resolution (~2 Å) in the postfusion conformation^21^, revealing substantial domain rearrangements between states, which is required of viral fusogens to mediate membrane fusion^29^. The fusion loops are located in DI, with the remaining domains playing distinct roles during the viral entry process^30^. Cryo-electron tomography had previously identified the structure of a membrane-bound HSV-1 gB partially stabilized in its prefusion conformation by an H516P substitution^28^. Based on this, we designed and assessed the effects of various alterations with the potential to stabilize the prefusion conformation of the gB ectodomain (Fig. 1). First, we characterized the impact of H516P in the context of a soluble ectodomain with an appended carboxy (C)-terminal phage T4 trimerization domain (foldon)^31^ (Fig. 1a, left panel). We observed the H516P ectodomain gB with an appended foldon to assume the postfusion form under negative-stain electron microscopy, with single-particle cryogenic electron microscopy (cryo-EM) revealing that although the DIII helices were interrupted at residue 516, the H516P ectodomain with an appended foldon was nonetheless in the postfusion conformation (Fig. 1c (left panel), Extended Data Fig. 1 and Supplementary Fig. 1**)**. This indicated that the H516P substitution did not impart sufficient postfusion destabilization to maintain the prefusion form in the absence of membrane. To provide additional postfusion destabilization, we introduced electro-repulsive charges via glutamate substitutions in the conformationally mobile DIII region to prevent formation of the postfusion DIII helical bundle (Fig. 1a, middle panel). Of five tested mutations causing alterations to glutamate, only L531E expressed (Supplementary Table 1). Negative-stain electron microscopy indicated that the H516P + L531E ectodomain (hereafter gB-Ecto.H516P.L531E) was in the prefusion conformation, and a single-particle cryo-EM structure at 3.7-Å resolution revealed L531E to be separated from other trimer equivalents by a Cγ distance of 9.3 versus 6.2 Å for the naturally occurring leucine in the postfusion conformation (Fig. 1c (middle panel), Extended Data Fig. 1 and Supplementary Fig. 1). Lastly, we introduced cysteine pairs into the interfaces between domains DI and DIV and between domains DI and DV to further stabilize the configuration of these mobile domains in the prefusion conformation (Fig. 1a, right panel). Of the nine tested potential disulfide (DS)-containing constructs, only A240C+E607C expressed (Supplementary Table 1).

Negative-stain electron microscopy indicated that gB-Ecto.H516P.L531E.DS was in the prefusion conformation, and the single-particle cryo-EM-based structure at 3.0-Å resolution revealed formation of the A240C–E607C disulfide bond locking domains DI and DIV in their characteristic neighbouring prefusion configuration (Fig. 1c (right panel), Extended Data Fig. 1a and Supplementary Fig. 1). We observed that the antigenicity of each ectodomain stabilized in the prefusion conformation was indistinguishable from that of postfusion gB when assessed by surface plasmon resonance (SPR) against the HSV-1 co-receptor, paired immunoglobulin-like type 2 receptor alpha (PILRα)^32^, and a panel of monoclonal antibodies^30,33,34^ (Extended Data Fig. 1b).

**Fig. 2:**
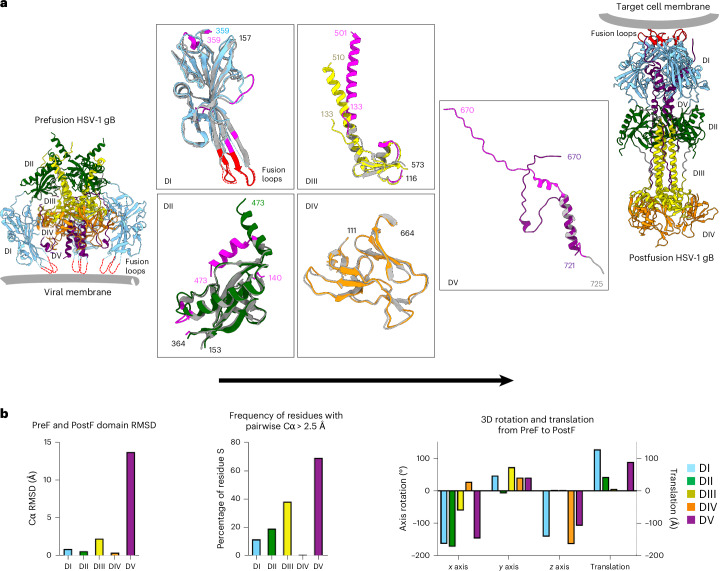
HSV-1 gB stabilized in the prefusion conformation, at 3-Å resolution, defines domain-based conformational change. **a**, Graphical depiction of domain-based conformational change during fusion, with boxed panels depicting the structural alignments of pre- and postfusion domains. Here the Fig. 1 domain colouring (blue, green, yellow, orange and purple, respectively, for DI, DII, DIII, DIV and DV) is preserved for prefusion gB, whereas postfusion gB is coloured grey, with fusion loops highlighted in red and postfusion residues with an RMSD of >2.5 Å highlighted in magenta. The N and C termini of continuous residues in each domain are numbered. The residue numbering is colour coded according to the structures, unless the residues are superimposed in both pre- and postfusion conformations, in which case they are labelled in black. In the prefusion structure, DI residues 174–184 and 252–266 (encompassing each of the fusion loops) and DV residues 722–725 are disordered. **b**, Quantification of conformational rearrangements by domain for the prefusion (PreF) to postfusion (PostF) transition of gB. Source data

**Extended Data Fig. 2:**
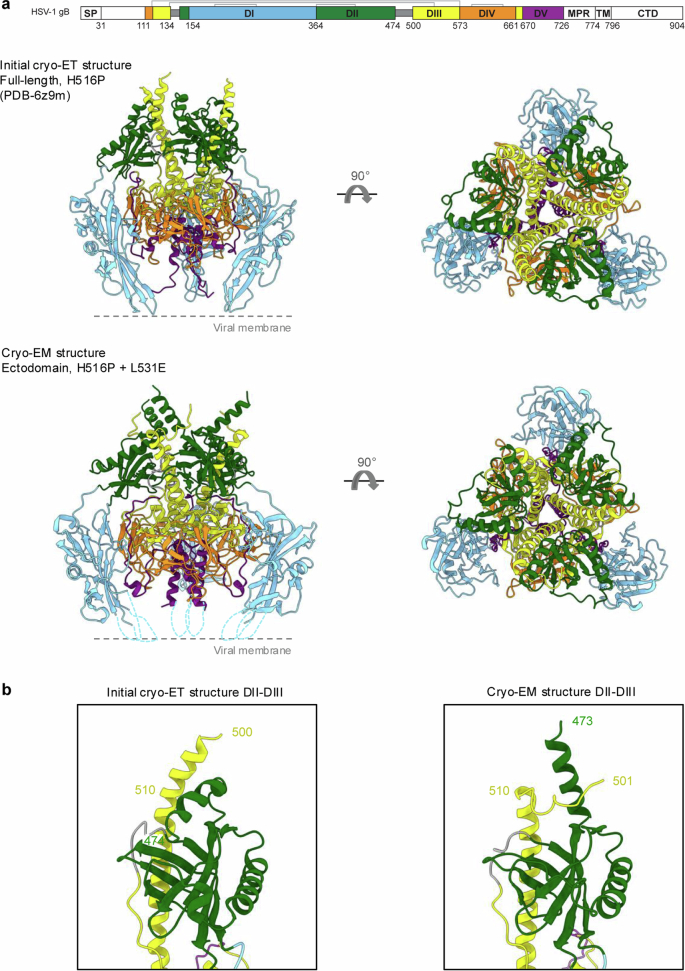
Comparison of prefusion HSV-1 gB structures. **a**, Orthogonal views of the overall prefusion HSV-1 gB trimer ectodomain structure as determined previously by cryo-ET of full-length, membrane-bound gB bearing H516P (top; PDB-6z9m)^27^ and in this study by single-particle cryo-EM of soluble gB ectodomain bearing H516P and L531E (bottom). Highly flexible and disordered regions of DI in the soluble ectodomain structure, which primarily encompass the fusion loops, are depicted with dashed lines. **b**, Comparison of interdomain interactions of the gB apex in the cryo-ET and cryo-EM structures, which represent the most notable structural differences between these two models. The high-resolution data presented in this study reveal the N-terminus of prefusion DIII to form a random coil that wraps around around the outwardly extended C-terminal helix of domain two (right), rather than the previously modelled compacted DII and fully elongated DIII helix (left) that more closely resemble features of the postfusion structure.

Most of the 3.0-Å-resolved cryo-EM reconstruction of prefusion gB closely resembled the structure modelled using cryo-electron tomography^28^, with the primary structural differences occurring at domain boundaries, such as between residues 501 and 510 at the amino (N)-terminal portion of DIII, where the prefusion structure assumed a random coil that wrapped around the extended C-terminal helix of DII, instead of an extended helix (Extended Data Fig. 2). Notably, other than DV, which refolded substantially, domains DI, DII, DIII and DIV (that is, DI–DIV) each rearranged primarily by rigid-body movements, with residues with >2.5-Å deviation between prefusion and postfusion conformations after domain superposition restricted to loops or to domain termini (Fig. 2a). For prefusion DI, the fusion loops (174–184 and 252–266) should be associated with the viral membrane, but were disordered and not visible in the reconstruction map of the prefusion ectodomain. Virtually all other elements superimposed well with the postfusion conformation, in which the fusion loops were associated with the target-cell membrane. For prefusion DII, residues 462–473 formed a helix that extended outward from the rest of the domain and pointed away from the viral membrane, but reoriented approximately 90° to form a compacted DII in postfusion gB. For DIII, as indicated above, postfusion residues 501–509 formed an N-terminal helix that was absent in the prefusion form, which then converged with a prefusion helix starting at residue 510; the overall DIII Cα root mean square deviation (RMSD) at 2.28 Å was higher than those for domains DI, DII and DIV because of the N-terminal helical diversion. For DIV, the pre- and postfusion domains were essentially identical, and indeed we used the prefusion location of domain DIV as a reference for movement. For prefusion DV, the domain RMSD was 12.2 Å, underscoring its substantial refolding. Thus, domains DI–DIV each transitioned between pre- and postfusion primarily en bloc, with substantial structural rearrangements occurring only in DV (Fig. 2b and Supplementary Table 2). This type of structural rearrangement suggested that there could be substantial plasticity in the pre- to postfusion movements of domains DI–DIV, as the movements occurred essentially with linked independent domains. Overall, structure-based design succeeded in stabilizing a soluble ectodomain in the prefusion conformation, with gB-Ecto.H516P.L531E stabilized and the disulfide-containing gB-Ecto.H516P.L531E.DS further stabilized by a covalent bond, with high-resolution details defining the domain-based pre- to postfusion conformational rearrangements of gB.

### Immunogenicity of prefusion HSV-1 gB

**Fig. 3:**
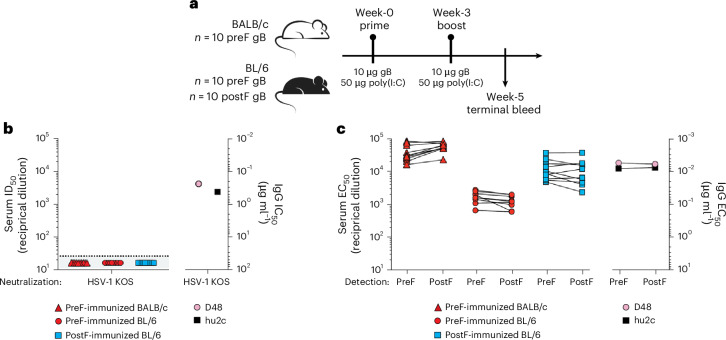
Prefusion HSV-1 gB immunization elicits equal pre- and postfusion reactivity, with no neutralization. **a**, Schematic of the immunization and readout regimen. **b**, Neutralization of authentic HSV-1 KOS strain. Left, ID_50_ values (that is, the reciprocal serum dilution that neutralized 50% of input virus) for the neutralization of week-5 mouse sera from the experimental groups defined in **a**. Sera samples (*n* = 10 per group) were tested with technical triplicates. Each data point corresponds to the average of technical triplicates for an individual sample. The limit of detection for the assay is designated with a horizontal dashed line. Right, IC_50_ values (that is, the concentration of antibody that neutralized 50% of input virus) for neutralization of the monoclonal antibodies D48 and hu2c, as determined using technical triplicates. The respective data points correspond to the average of technical triplicates for each antibody. **c**, ELISA binding to gB. Left, ELISA EC_50_ titres of week-5 mouse serum recognition of prefusion (gB-Ecto.H516P.L531E.DS) and postfusion (gB-Ecto.H516P) gB. Serum samples (*n* = 10 per group) were tested with technical duplicates. Each data point corresponds to the average of technical duplicates for an individual sample. Right, ELISA EC_50_ titres for the monoclonal antibodies D48 and hu2c binding to pre- and postfusion gB, as determined using technical duplicates. The respective data points correspond to the average of technical duplicates for each antibody. The higher-titre EC_50_ responses in BALB/c relative to BL/6 mice are expected since BALB/c mice skew towards type 2 helper T cell innate immunity, leading to stronger humoral responses. Source data

**Extended Data Fig. 3:**
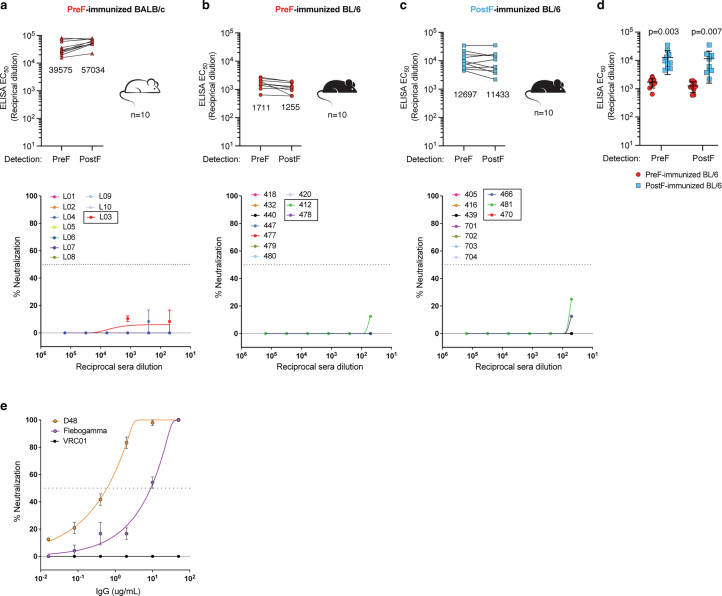
Detection of HSV-1 gB binding and HSV-1 neutralizing responses. **a-c**, ELISA binding (top) and authentic HSV-1 KOS neutralization (bottom) by week 5 sera from immunized mice, divided by immunization group (*n* = 10 per group). Immunogen and mouse strain are listed at the top of each column. Top, mean ELISA EC_50_ values for each conformation of gB are listed under each set of data, with each data point indicating the mean value from two technical replicates. Bottom, mice with low levels of detectable neutralization (reduction of cytopathic effect; CPE) are boxed: one preF-immunized BALB/c, two preF-immunized BL/6, and three postF-immunized BL/6. Each point represents the mean value of technical triplicates with error bars depicting the standard deviation. **d**, Direct comparison of ELISA EC_50_ titres elicited in BL/6 mice to highlight that postF gB is more immunogenic than preF gB. Error bars depict standard deviation, together with a measure of the mean for each group. Statistical differences were determined by two-tailed Student’s *t*-test. **e**, Neutralization of authentic HSV-1 KOS by monoclonal antibody D48 and the pooled human IgG reagent, Flebogamma. VRC01 is an HIV-neutralizing monoclonal antibody used as a negative control. Each point represents the mean value of technical triplicates, with error bars depicting the standard deviation. Source data

The prefusion conformational stability of gB-Ecto.H516P.L531E.DS enabled us to assess the immunogenicity of the soluble prefusion gB ectodomain. Specifically, we immunized BALB/c mice at 0 and 3 weeks with 10 µg gB-Ecto.H516P.L531E.DS in 50 µg poly(I:C) adjuvant and measured the ability of week-5 sera to neutralize HSV-1 (Fig. 3a and Extended Data Fig. 3). Although the previously described gB-directed antibodies hu2c (refs. ^35,36^) and D48 (ref. ^27^) showed potent neutralization of the HSV-1 KOS strain (Fig. 3b), no neutralization was observed in any of the ten immunized BALB/c mice (Fig. 3b). To test whether this lack of neutralization related to the mouse strain tested, we further assessed both pre- and postfusion gB ectodomains in BL/6 mice. Again, no HSV-1 neutralization was observed (Fig. 3b). Despite the lack of neutralization, each of the 30 mice showed essentially equivalent enzyme-linked immunosorbent assay (ELISA) titres to pre- and postfusion gB. For the ten BALB/c mice immunized with gB stabilized in the prefusion conformation, pre- and postfusion titres showed half-maximal binding around 10^4^–10^5^ dilution; for the ten BL/6 mice immunized with postfusion gB, pre- and postfusion titres were around 10^4^; and for the ten BL/6 mice immunized with gB stabilized in the prefusion conformation, pre- and postfusion titres were around 10^3^–10^4^ dilution (Fig. 3c). Thus, immunization with either pre- or postfusion gB yielded substantial binding titres to both pre- and postfusion gB that were essentially equivalent, with no measurable HSV-1 neutralization.

### Iso-surface accessibility, hydrogen–deuterium exchange/mass spectrometry and refolding analysis of gB

**Fig. 4:**
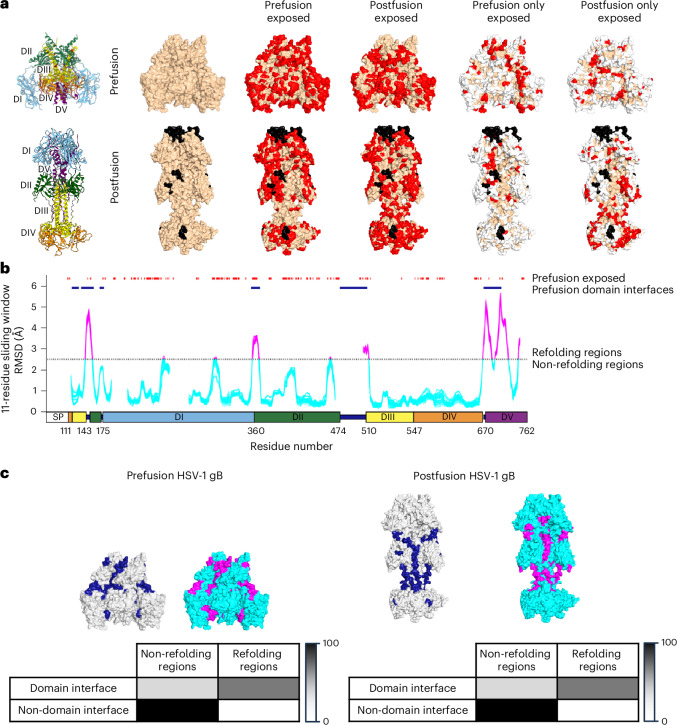
Virtually all accessible surfaces on prefusion HSV-1 gB are present on postfusion gB, with refolding regions segregated to domain interfaces. **a**, Accessible-surface analysis indicating iso-surface exposure. Accessible surfaces are shown in red. White represents surfaces exposed in both conformations and black represents residues missing in the structure of the alternative conformation. Virtually all exposed surfaces on prefusion HSV-1 gB are present on postfusion gB. A probe radius of 5 Å was used to better simulate recognition by an antibody combining site. **b**, Identification of refolding regions (magenta) and domain interfaces (dark blue), showing that refolding prefusion surfaces are relegated to domain interfaces. A refolding RMSD of 2.5 Å was selected as changes of this magnitude should substantially impact antibody recognition. **c**, Correspondence between domain interfaces and refolding regions. Molecular models of pre- and postfusion HSV-1 gB are shown on the left and right, respectively. The heatmaps show the fractions of total domain interface residues (blue) and non-domain interface residues (row totals) located in non-refolding (cyan) or refolding regions (magenta). The heatmaps are coloured from white (0% of residues) to black (100% of residues). The colours used for molecular surfaces are coordinated to match the features of the heatmap. The numbers of domain interface residues were 20 in non-refolding regions and 47 in refolding regions. The numbers of non-domain interface residues were 460 in non-refolding regions and 33 in refolding regions. Of note, domain interfaces had a tenfold greater proportion of refolding regions than non-domain interfaces (70% versus 7%). Source data

To provide insight into the similarity in binding titres between pre- and postfusion gB and the observed lack of neutralization elicited by gB immunization, we used a 5-Å probe radius to simulate the accessibility of antibody-combining loops, and analysed accessible surfaces of prefusion gB for their location in postfusion gB; notably, we observed iso-surface exposure, with virtually all of the accessible surfaces on prefusion gB similarly exposed on postfusion gB (Fig. 4a). Indeed, only the DI-occluded surfaces on prefusion domains DIII and DIV were newly exposed in the postfusion conformation. Analysis of postfusion gB yielded similar results (Fig. 4a). Thus, virtually all exposed and accessible surfaces on prefusion gB were also exposed and accessible in postfusion gB, with conformation-unique exposed surfaces relegated primarily to domains DIII and DIV, which DI occludes in the prefusion conformation.

**Extended Data Fig. 4:**
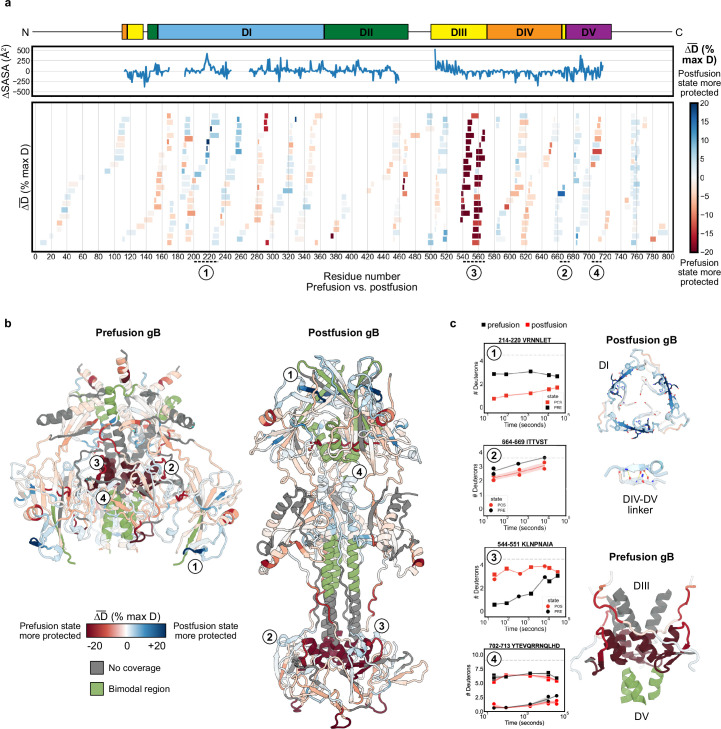
Hydrogen-deuterium exchange/mass spectrometry (HDX) analysis of prefusion-stabilized and postfusion gB ectodomains. **a**, Peptide coverage and differential hydrogen-deuterium exchange for prefusion and postfusion gB ectodomains determined by mass spectroscopy analysis. The regions are coloured according to the legend for relative protection in either conformation: red regions are more protected from hydrogen-deuterium exchange in prefusion gB, and blue regions are more protected in postfusion gB. **b**, Mapping of hydrogen-deuterium exchange differences onto prefusion (left) and postfusion (right) structures to highlight conformational changes. **c**, Representative uptake plots for the numbered regions in **a** and **b**, with structural details on the right. These regions exchange differently in the two gB states. Peptide 4 has bimodal exchange behaviour, suggesting two conformations in solution. *n* = 2 technical replicates (shapes). See Supplementary Figs. 2-4 for additional information on HDX/MS experiments and analysis. Source data

To provide further insight into the surface chemistries of pre- and postfusion gB, we performed hydrogen–deuterium exchange/mass spectrometry (HDX) comparing the differences in exchange for both conformations of gB (Extended Data Fig. 4, Supplementary Figs. 2–4 and Supplementary Table 3). The largest differences in exchange were observed at residues 537–568, in DIII, where the local domain folding is similar, but the interactions between domains differed due to the rearrangement of domain orientations; specifically, we observed increased protection from hydrogen exchange in DIII of prefusion gB compared with the postfusion state due to increased interactions within the domain, as well as increased protection in regions of DV and DIV from increased interactions with DIII. Interestingly, we identified three regions of gB with bimodal distributions of hydrogen exchange, suggestive of distinct conformations that were sampled during the timescale of this experiment: a DV refolding region (residues 700–716) in both pre- and postfusion gB; and two trimer interface regions (residues 164–178 and 506–521) in postfusion gB (Extended Data Fig. 4b,c and Supplementary Fig. 4). Overall, despite conformational reorganization of gB proceeding in an iso-surface manner such that virtually all exposed and accessible surfaces of prefusion gB were present on postfusion gB, we observed clear differences in HDX between pre- and postfusion gB.

To provide explicit quantification of gB refolding, we identified refolding regions by evaluating structural differences between pre- and postfusion conformations using a 11-amino-acid sliding window^37^. Other than DV, only a few short segments showed RMSDs above 2.5 Å (Fig. 4b). These resided: at the DII–DIII interface (residues 132–142), which transitions from a bent connection (18.2 Å in length) in prefusion gB, in which only residues 136 and 141 are exposed (with 141 being a site of *N*-linked glycosylation), to a straight connection (30.0 Å) in postfusion gB, which is mostly exposed; at the DI–DII interface (residues 358–366), which transitions from an extended strand (20.8 Å) in prefusion gB to a more compact strand with one turn (17.4 Å) in postfusion gB, of which only residues 158, 360 and 361 are exposed; and at the DII–DIII interface (resides 506–513), which is part strand and part helix in the prefusion state (with resides 505, 509, 511 and 512 being exposed) and fully helical in the postfusion state. Notably, these refolding regions generally corresponded to domain interfaces (Fig. 4c). Consistent with the refolding and accessibility analyses, the HDX experiments showed increased protection in prefusion gB for the refolding regions (Extended Data Fig. 4 and Supplementary Figs. 2–4). In contrast, DV showed substantial structural rearrangements and was mostly occluded within the trimer. Thus, refolding regions between pre- and postfusion gB often corresponded to domain interfaces that were occluded and inaccessible for antibody recognition.

### Glycan shielding of gB

**Fig. 5:**
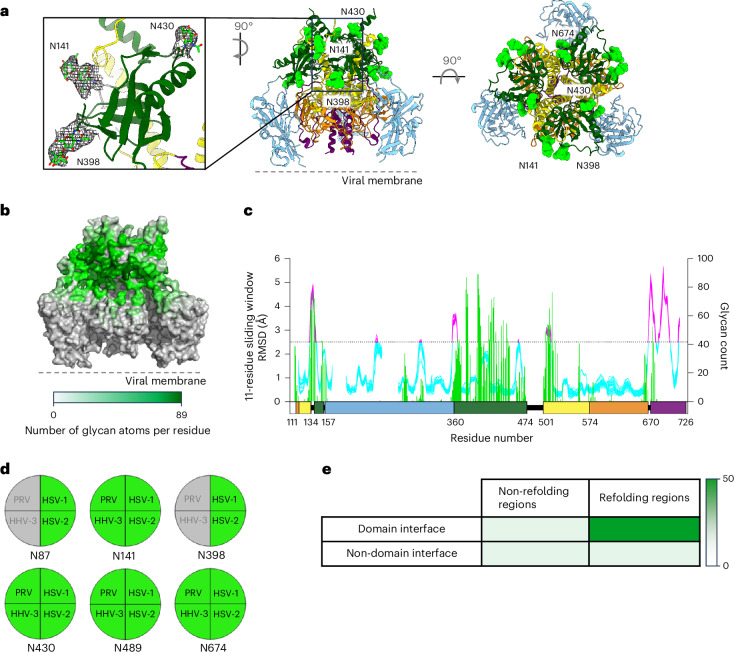
Glycan shielding of the gB apex, leaving DI and DIV unencumbered. **a**, Identification of *N*-linked glycans on the prefusion gB structure, highlighting the cryo-EM reconstruction density for several of these glycans. Labels are provided for glycans on a single protomer. **b**,**c**, Prefusion gB *N*-linked glycan coverage, as determined by molecular dynamics simulation, depicted by structure (**b**) and sequence (**c**). In c, green, magenta and cyan represent glycan shielding, refolding regions and non-refolding regions, respectively. *N*-linked glycans shield the gB apex and occlude refolding regions outside of DV. **d**, Sites of *N*-linked glycosylation in HSV-1 gB, coloured green if conserved or grey if absent in three other common alphaherpesviruses: HSV-2, HHV-3 and pseudorabies virus (PRV). **e**, Heatmap showing the degree of total residue masking related to residue-level correspondence between refolding regions, surface exposure and glycan shielding. The heatmap is coloured from white (0% of total residues are masked) to dark green (50% of total residues are masked). For domain interface residues, three of 20 (15%) are shielded in non-refolding regions and 23 of 47 (49%) are shielded in refolding regions. For non-domain interface residues, 79 of 460 (17%) are shielded in non-refolding regions and five of 33 (15%) are shielded in refolding regions. Of note, refolding regions at domain interfaces are the most highly masked by glycans. Source data

HSV-1 gB has six potential sites of *N*-linked glycosylation—at residues 87, 141, 398, 430, 489 and 674. The densities for 141, 398, 430 and 674 were visible in the reconstruction (Fig. 5a). For most glycans, the density was clear for only the protein-proximal *N*-acetylglucosamines; the lack of density for other saccharides probably resulted from glycan flexibility.

**Extended Data Fig. 5:**
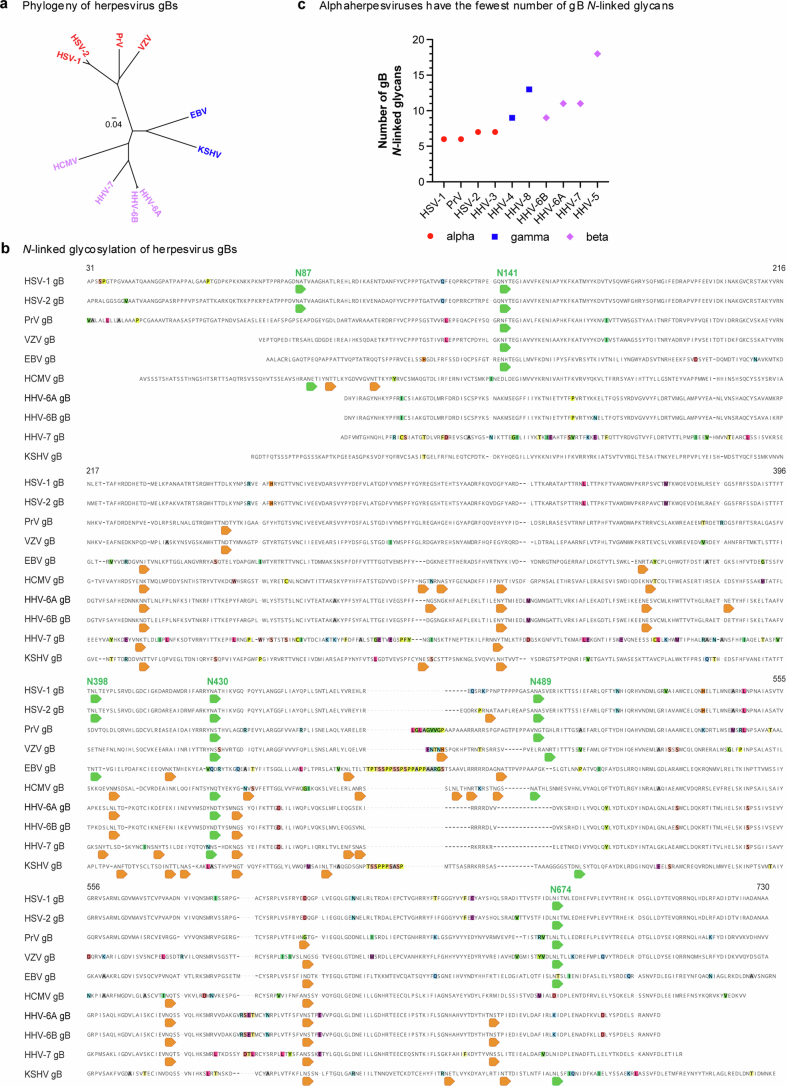
Glycan shielding of gB is conserved across human herpesviruses. **a**, Amino acid maximum likelihood phylogeny of gB from common alphaherpesviruses (red), betaherpesviruses (purple), and gammaherpesviruses (blue). **b**, Amino acid alignment of herpesvirus gB sequences with potential *N*-linked glycosylation sites highlighted in green if they are conserved in HSV-1 and orange if not conserved in HSV-1. **c**, Graphical quantification of potential *N*-linked glycosylation sites.

**Extended Data Fig. 6:**
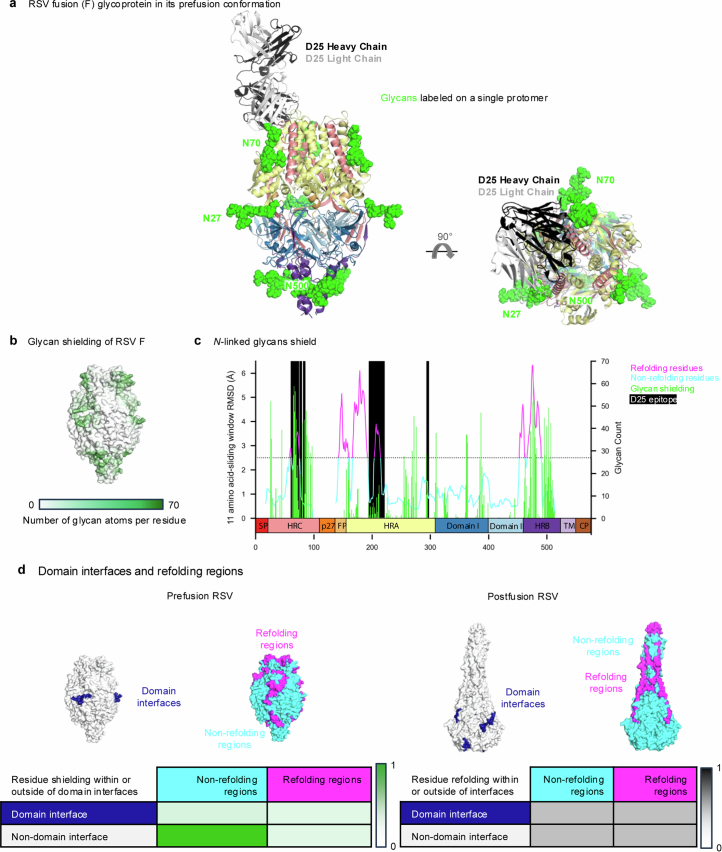
Accessible surface and refolding analysis of RSV F. **a**, Structure of trimeric RSV F ectodomain with a single D25 Fab. **b**, Prefusion RSV F coloured by *N*-linked glycan coverage determined through molecular dynamics simulation. **c**, Residue-specific quantification of the RSV F surface accessibility, refolding (using a 9-residue sliding window), and glycan shielding, with the D25 epitope highlighted (the D25 epitope contained 31 residues in the HRC and HRA domains). **d**, Domain interfaces and refolding regions are shown for prefusion (left) and postfusion (right) conformations of RSV F. Residue-level correspondence is shown between refolding regions, surface exposure, and glycan shielding. Source data

All visualized glycans were in the top half of the trimer, and a per-atom quantification of glycan shielding revealed much of the trimer apex to be covered or proximal to *N*-linked glycan (Fig. 5b). Notably, many of the refolding regions were masked by glycan (Fig. 5c). Moreover, HSV-1 gB glycans were mostly conserved in alphaherpesviruses (Fig. 5d and Extended Data Fig. 5). The membrane-distal apex of prefusion gB was substantially shielded by glycan, whereas the membrane-proximal DI and DIV were glycan free. Overall, refolding regions in domains DI, DII, DIII and DIV were at domain interfaces and occluded, with substantial glycan shielding; indeed, only two domain interface residues in DI through DIV refolded, were exposed and were free of glycan shielding (residues 357 and 358), and only three non-domain interface residues in DI though DIV refolded, were exposed and were free of glycan shielding (306, 309 and 462) (Fig. 5e). We note that the occlusion of refolding residues in HSV-1 gB contrasts with RSV class I fusion glycoprotein F, where a cluster of refolded residues (residues 196–219) are exposed and free of glycan shielding at the binding site of neutralizing antibody D25 (ref. ^12^) (Extended Data Fig. 6a–c). In addition, unlike HSV-1 gB, RSV glycoprotein F exhibited little correspondence between refolding regions and domain interfaces (Extended Data Fig. 6d).

### Cryo-EM structures of the antibodies WS.HSV-1.24 and D48

**Extended Data Fig. 7:**
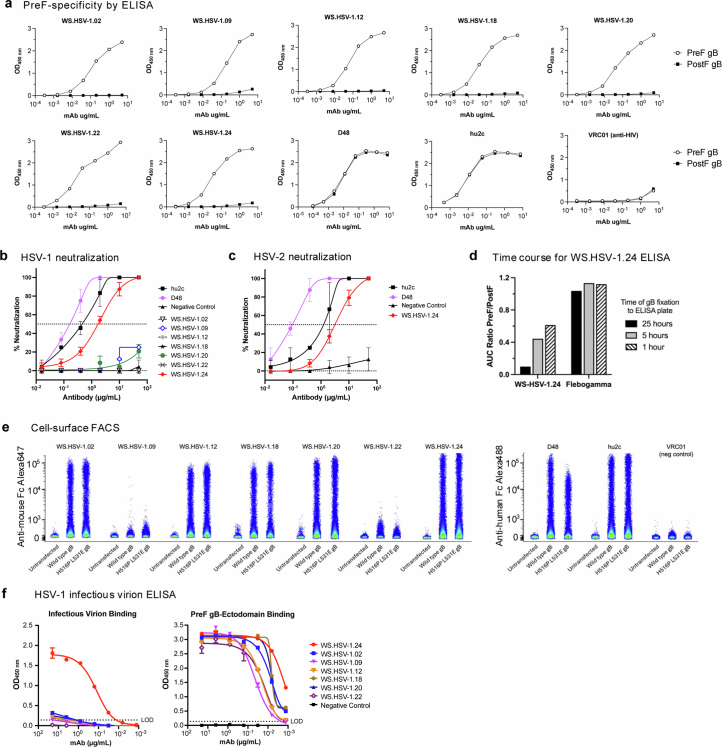
Prefusion-specific HSV-1 antibodies: immunogenetics, neutralization, and binding. **a**, ELISA antibody recognition of prefusion (gB-Ecto.H516P.L531E.DS) and postfusion (gB-Ecto.H516P) gB. Each data point indicates the mean value from two technical replicates **b**, Authentic HSV-1 KOS neutralization by murine antibodies and monoclonal human antibodies D48 and hu2c. Each data point represents the mean value of technical triplicates with error bars depicting the standard deviation. **c**, Authentic HSV-2 neutralization by monoclonal human antibodies D48 and hu2c and murine antibody WS.HSV-1.24. Each data point represents the mean value of technical triplicates with error bars depicting the standard deviation. **d**, AUC ratio of time course for WS.HSV-1.24 ELISA in which gB was coated on plates for the indicated amount of time. **e**, Cell-surface FACS of full-length wild type and prefusion-stabilized HSV-1 gB. **f**, Infectious HSV-1 virion ELISA and soluble gB ELISA control assay. Each data point represents the mean value of technical triplicates with error bars depicting the standard deviation. Source data

To determine whether gB-directed neutralizing antibodies had been induced (despite the absence of serum neutralization), we generated B cell hybridomas from the splenocytes of prefusion gB-Ecto.H516P.L531E.DS-immunized BALB/c mice (screened by ELISAs for the recognition of pre- but not postfusion gB) and identified and sequenced immunoglobulin transcripts, which were then expressed as monoclonal antibodies and tested for neutralization (Extended Data Fig. 7a–c and Supplementary Table 4). One antibody, WS.HSV-1.24, neutralized both HSV-1 and HSV-2 (Extended Data Fig. 7b,c). Although the gB binding of these antibodies differed in surface plasmon resonance and ELISA contexts (Extended Data Fig. 7a,d and Supplementary Table 5) and several retained binding in a cell-surface context (Extended Data Fig. 7e and Supplementary Fig. 6), WS.HSV-1.24 was the only antibody that bound infectious HSV-1 virions (Extended Data Fig. 7f). Other antibodies that did not neutralize had lower gB affinity and their binding may have been occluded by other viral glycoproteins^38^.

**Fig. 6:**
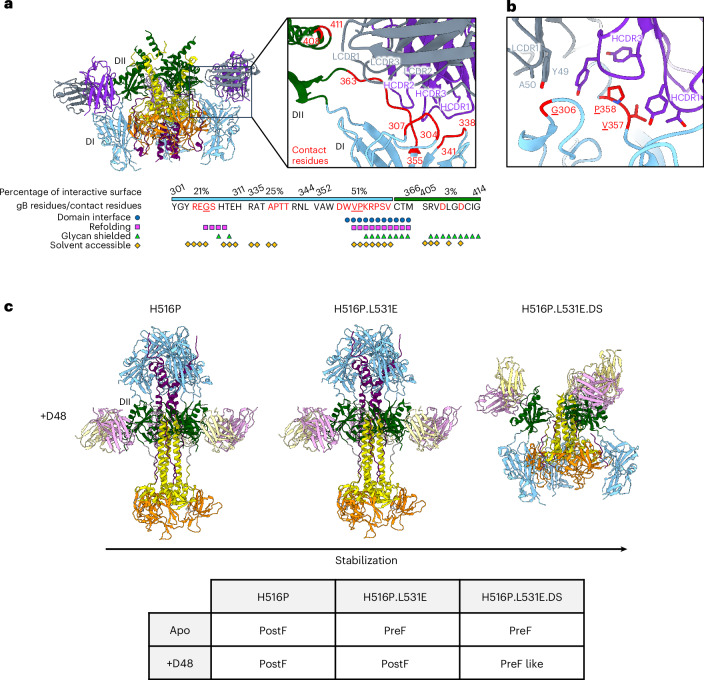
Cryo-EM structures of the antibodies WS.HSV-1.24 and D48 in complex with HSV-1 gB reveal mechanisms of gB neutralization. **a**, Structure (left) and expanded view (right) of the interaction of prefusion-specific Fab WS.HSV-1.24 with HSV-1 gB-Ecto.H516P.L531E. Select features of WS.HSV-1.24 contact residues (labelled red throughout) are highlighted below. Heavy- and light-chain WS.HSV-1.24 and their specific residue labels are shown in purple and grey, respectively. DI and DII are shown in blue and green, respectively. **b**, WS.HSV-1.24 recognition of refolding, solvent-exposed, non-glycan-shielded residues. The underlined residues correspond to those in the bottom panel of **a**. **c**, Structures of Fab D48 in complex with three different gB-Ecto variants (top) and a matrix of the molecular phenotypes (bottom). Light- and heavy-chain D48 are shown in yellow and pink, respectively.

**Extended Data Fig. 8:**
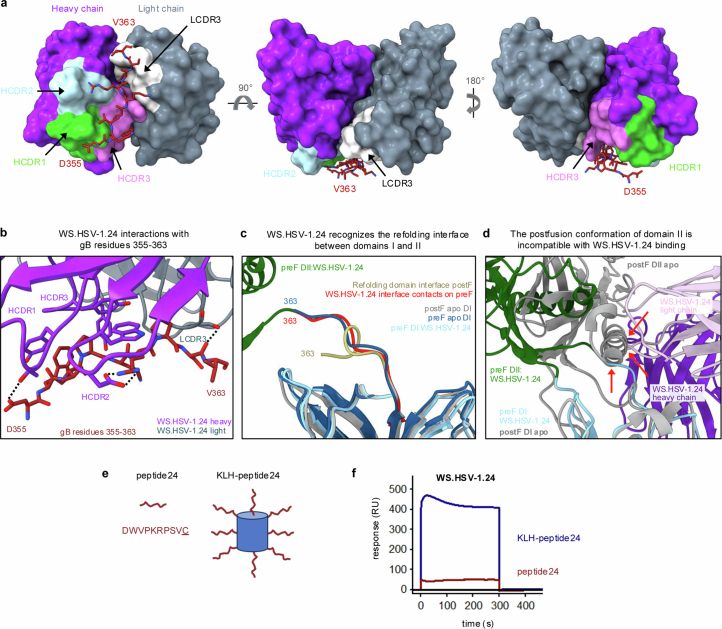
Details for cryo-EM structure of WS.HSV-1.24 in complex with gB-Ecto.H516P.L531E and WS.HSV-1.24 epitope-focused vaccine design. **a**, Orthogonal views of the linear peptide corresponding to the DI-DII refolding domain interface (gB residues 355-363, coloured dark red and shown in stick representation) bound to WS.HSV-1.24 (shown in surface representation) to highlight CDR recognition. Heavy and light chain CDRs that comprise the paratope and are labelled and coloured distinctly. **b**, Residue-level interactions of WS.HSV-1.24 with the linear domain interface between DI and DII (gB residues 355-363). **c**, Comparison of the C-terminal refolding interface of domains DI and DII between apo prefusion gB, WS.HSV-24-bound prefusion gB, and apo postfusion gB. **d**, Alignment of DI from apo postfusion gB and WS.HSV-24-bound prefusion gB structures, revealing several putative clashes (denoted by red arrows) with the orientation of postfusion DII that demonstrates the postfusion conformation of gB to be incompatible with WS.HSV-24 recognition. **e**, Proteins used for SPR binding analysis. Peptide24 corresponds to the refolding interface between domains DI and DII that is recognized by WS.HSV-1.24, with the addition of a C-terminal cysteine. KLH-peptide24 was prepared by conjugating the free sulfhydryl group on the C-terminal cysteine of peptide24 to maleimide-activated KLH. **f**, SPR curves of peptide24 and KLH-peptide24 binding to WS.HSV-1.24.

We determined the single-particle cryo-EM structure of the antigen-binding fragment (Fab) of WS.HSV-1.24 recognizing gB-Ecto.H516P.L531E (Fig. 6a and Supplementary Fig. 7). WS.HSV-1.24 bound at the DI–DII interface to three (306, 357 and 358) of the seven gB trimer residues that our analysis revealed to be refolding, exposed and not occluded by glycan (Fig. 6b and Extended Data Fig. 8). Thus, the prefusion-specific WS.HSV-1.24 antibody bound an epitope comprising primarily the interface between domains DI and DII, as well as surface loops from both domains. The prefusion-specific epitope encompassed refolding residues that were not exposed or were glycan shielded, suggesting the epitope to be immune recessive, consistent with the observation of no neutralization in the donor mouse serum.

We also analysed antibody D48, which has been shown to recognize gB and prevent entry^27,39^. We determined structures by single-particle cryo-EM of D48 Fab in complex with each of the three versions of stabilized HSV-1 gB that we had designed (Fig. 6c and Supplementary Fig. 8). With the complex of D48 Fab with gB-Ecto.H516P (3.0-Å resolution), the D48 Fab-recognized gB was in the postfusion conformation (Fig. 6c, left). With the complex of D48 Fab with gB-Ecto.H516P.L531E (3.8-Å resolution), D48 Fab induced the postfusion conformation (Fig. 6c, middle). When D48 Fab was complexed with gB-Ecto.H516P.L531E.DS, a hybrid/distorted pre-like structure was observed (4.1-Å resolution; Fig. 6c, right).

**Extended Data Fig. 9:**
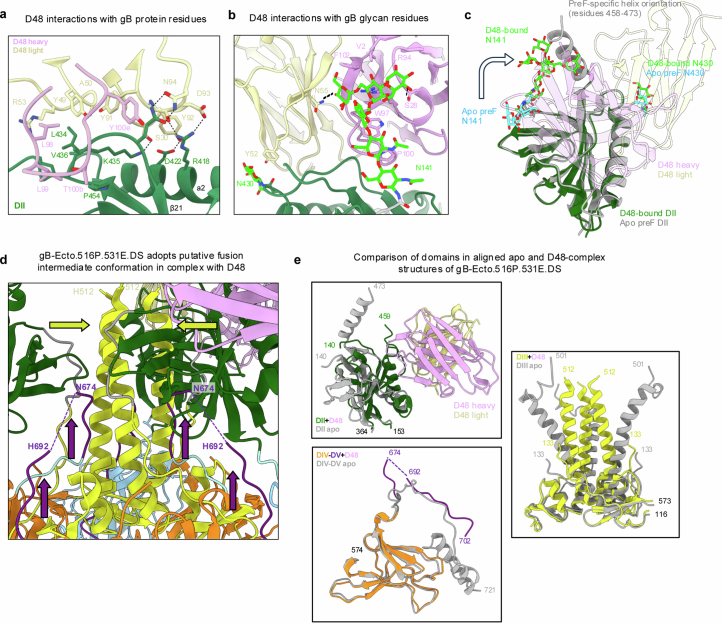
Details for cryo-EM structures of D48 in complex with gB. **a**,**b**, Expanded views of the interaction between D48 and postfusion gB amino acids (**a**) and glycans (**b**). **c**, Visualization of the clash between the D48-enforced conformation of glycan 141 and the prefusion-specific orientation of the helical residues 458-473. **d**, Highlighting features of the putative fusion-intermediate structure captured by D48 in complex with DS-stabilized gB, including formation of the DIII helical bundle and rearrangement of DV. The arrows indicate the direction of conformational change for DIII and DV from apo prefusion gB. **e**, Comparison of gB-Ecto.516 P.531E.DS domains DII, DIII and D1V-DV from the aligned prefusion apo (grey) and D48 complex (coloured) structures. Trimeric DIII is shown to emphasize the different conformations of the two structures. Notably, a majority of DV has become disordered in the in the D48 complex.

Examination of D48 Fab–gB-Ecto.H516P and D48 Fab–gB-Ecto.H516P.L531E structures revealed D48 to straddle the 432–440 loop connecting the α2 helix and β21 strand, with recognition of *N*-linked glycans N141 and N430 contributing 42% of the interactive surface (Extended Data Fig. 9a,b). The orientation of glycan N141 enforced by D48 binding would sterically clash with the prefusion-specific configuration of the C-terminal DII helix (residues 460–474) (Extended Data Fig. 9c). The fact that D48 induced the postfusion conformation in gB-Ecto.H516P.L531E (which in the absence of D48 exclusively assumed a prefusion conformation) suggested that D48 functions by prematurely inducing the postfusion conformation of gB. In addition, examination of the D48 Fab–gB-Ecto.H516P.L531E.DS ectodomain structure revealed D48 to capture the disulfide-stabilized gB with a displaced C-terminal DII helix, with formation of the DIII helical bundle and with an upward rearrangement of DV, potentially reflecting an intermediate state between pre- and postfusion conformations (Fig. 6c and Extended Data Fig. 9d,e).

## Discussion

In this study, we demonstrate that immunization with gB stabilized in the prefusion conformation elicits high binding titres, but no neutralization of HSV-1, and propose that gB evades neutralizing antibody responses by utilizing two gB features: (1) an iso-surface display of antibody-accessible surfaces for pre- and postfusion conformations; and (2) structural plasticity, enabling it to shift between conformations even in the presence of binding antibody. The latter is achieved through rigid-body rearrangement of surface-exposed domains, a type of movement that can occur with few constraints on the specific pathway of rearrangement. The proposed gB evasion differs from other types of evasion, such as glycan shielding, which prevents the elicitation of antibodies, with iso-surface plasticity instead preventing neutralization by binding antibodies that have already been elicited. Since we stabilized the gB ectodomain in the prefusion conformation, not as the full-length membrane-bound gB, certain regions were excluded (that is, the membrane-embedded MPR domain) or were left flexible and could not be resolved with high resolution (that is, the N-terminal region (residues 90–103) and fusion loops); nonetheless, our analyses extended to most of the surface-exposed gB trimer. Overall, our results provide insight into why the design strategy involving stabilization of the prefusion conformation of entry machines that has dominated the viral vaccine field does not apply to HSV-1.

Despite HSV-1 gB being mostly impervious to neutralization by antibodies recognizing its prefusion shape, a few gB-specific antibodies have been found to neutralize, including those that prevent gB from binding membrane bilayers^34,40^. In addition, immunization with peptide encoding the second fusion loop can elicit neutralizing antibodies, albeit with low titres^34^. Here we provide the molecular basis for the neutralization mechanism of antibody D48 through pre-triggering of the postfusion state (a type of neutralization observed with the severe acute respiratory syndrome coronavirus neutralizing antibody S230)^41^. We also identify a prefusion-specific antibody (WS.HSV-1.24) and show that it neutralizes by recognizing three of seven less masked gB refolding residues, two of which are located in the flexible refolding region between domains DI and DII. Overall, HSV-1 gB generally avoids prefusion neutralization, but can be neutralized by select antibodies that recognize critical features (for example, fusion loops), that recognize prefusion conformation-specific refolding regions (for example, WS.HSV-1.24) or that stabilize the postfusion state (for example, D48). Although the cryo-EM structures presented here provide strong evidence for the pre-triggering mechanism of D48, future investigation is needed to confirm that WS.HSV-1.24 recognition of prefusion gB can in fact induce conformational change.

We note that stabilization of the prefusion state has been the standard means of prefusion neutralization. This standard mechanism of neutralization is avoided by HSV-1, in part, through iso-surface plasticity, although recognition of refolding regions or binding across domains—both of which can be achieved by WS-HSV-1.24—can overcome this avoidance. However, refolding regions on gB are generally masked and immune recessive by a lack of surface exposure and/or glycan shielding, and binding across domains is generally rare (for example, it is observed in only 7 of the 703 severe acute respiratory syndrome coronavirus 2 antibody–spike complexes deposited in the Protein Data Bank (PDB)^42^. We note in this context that neutralization of the class III rabies virus entry machine by the antibody RVA122 also utilizes stabilization of the prefusion conformation, so class III prefusion-specific neutralization has been observed previously^43^, although with RVA122 recognition occurs at a large prefusion-specific multidomain surface at the rabies glycoprotein apex, not at occluded refolding interfaces as with WS.HSV-1.24.

**Extended Data Fig. 10:**
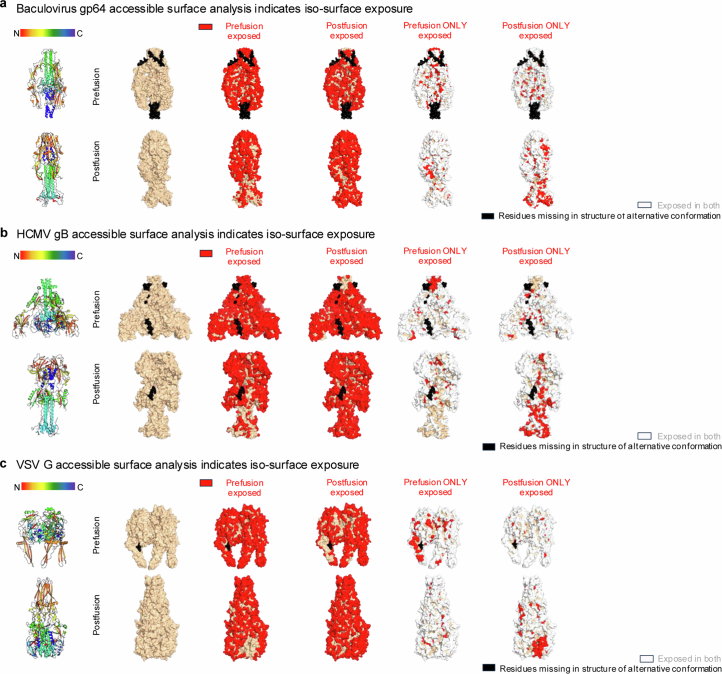
Iso-surface analysis of additional class III-fusion machines. **a-c**, Accessible surfaces (red) are displayed on prefusion and postfusion conformations of fusion machines from baculovirus (PDB IDs: 8YG6 and 3DUZ; **a**), HCMV (PDB IDs: 8VYN and 8VYM; **b**) and VSV (PDB IDs: 5I2S and 5I2M; **c**). A probe radius of 5 Å was used to better simulate recognition by an antibody combining site.

The high structural conservation of postfusion gBs across the nine different herpesviruses that infect humans suggests that their prefusion forms might be similarly ineffective at eliciting prefusion-specific neutralizing responses, although only a few prefusion gB structures are known, one of which is the gB of human cytomegalovirus (HCMV)^44,45^. Compared with the HSV-1 prefusion gB that we determine here, the prefusion structure of HCMV gB is highly similar, with the most prominent difference being open versus assembled DIII helical bundles; this suggests similar prefusion vaccine ineffectiveness—and indeed the recently reported prefusion conformation stabilization and immunization of HCMV gB also showed essentially no neutralization^45^. Moreover, we observed that HCMV, as well as class III fusion machines from baculovirus and vesicular stomatitis virus, displayed substantial iso-surfaces between pre- and postfusion conformations like HSV-1 gB (Extended Data Fig. 10 and Supplementary Table 6). Thus, although stabilization of the prefusion conformation is a vaccine strategy that has succeeded with multiple viral fusion machines, our results indicate that, for herpesviruses, prefusion gB stabilization may not be a viable vaccine strategy unless the immunogenicity of refolding regions—which we show to be masked in prefusion HSV-1 gB—can be substantially increased. One potential way to do this is through epitope focusing, as has been done with the HIV-1 fusion peptide site of vulnerability^46^ by first priming with a peptide conjugate corresponding to the recognized epitope and then boosting with prefusion trimers^47,48^; indeed, initial experiments demonstrate WS.HSV-1.24 to bind a nine-residue peptide corresponding to the recognized refolding domain interface (Extended Data Fig. 8e,f). Future studies will need to investigate whether epitope focusing or other structure-based vaccine design strategies can overcome iso-surface plasticity and induce effective WS.HSV-1.24-like neutralizing responses in animal models and, ultimately, humans. It will be interesting to determine how the in vivo protective efficacy of prefusion conformation-specific neutralizing antibodies compares with the efficacies of those antibodies that recognize both conformations of gB.

## Methods

### Ethics oversight

All of the experimental procedures and methods for mouse immunizations were conducted in compliance with the ethical regulations of Columbia University and the National Institutes of Health (NIH). All of the mouse experiments were reviewed and approved by the respective Institutional Animal Care and Use Committees of Columbia University (protocol AABE9556) and the Vaccine Research Center, National Institute of Allergy and Infectious Diseases, NIH (protocol VRC-22-0977).

### Design and production of stabilized gB

A codon-optimized gene encoding residues 1–728 of HSV-1 KOS-strain gB (GenBank: E03113.1) with the partial prefusion-conformation-stabilizing H516P substitution^28^, a C-terminal T4 fibritin trimerization domain, an HRV 3C protease cleavage site, an 8xHis-Tag and a Twin-Strep-tag was synthesized and cloned (GenScript) into the mammalian expression vector pαH. This design, termed gB-Ecto.H516P, served as the base construct for which all subsequent putative mutations, encoding alterations intended to stabilize the prefusion conformation of the protein, were introduced by site-directed mutagenesis using KOD Xtreme Hot Start DNA Polymerase (Sigma–Aldrich). Positions selected to introduce glutamate substitutions, to electrostatically destabilize the postfusion DIII helix bundle, were interface residues between H516 and E535 in the postfusion gB structure (PDB ID: 2GUM). Positions selected to introduce paired interdomain cysteine substitutions to form artificial disulfide bonds restricting the conformational rearrangement of DI were stretches of DIV and DV residues proximal to DI in the prefusion gB cryo-tomography structure (PDB ID: 6Z9M).

To express HSV-1 gB ectodomains, Expi293F cells were transiently transfected with plasmid DNA for each gB construct (1 µg DNA per ml culture) using PEI MAX at a 1:3 ratio (DNA:polyethylenimine (PEI)). Five days after transfection, cultures were clarified by centrifugation and harvested supernatants were filtered with a 0.45-µM polyethersulfone membrane. HSV-1 gB was first purified from culture supernatants using His60 Ni Superflow Resin (Takara Bio) with gravity flow, followed by Strep-Tactin XT 4Flow Resin (IBA Lifesciences) with gravity flow. For gB proteins that were used for structural and immunization studies, a final purification polishing step and buffer exchange into 10 mM HEPES and 150 mM NaCl (pH 7.5) was performed using gel filtration with a Superose 6 Increase 10/300 column.

### Production of antibodies and gB co-receptor

The heavy- and light-chain variable region genes of human antibodies (D48 (ref. ^27^) and hu2c^36^) and vaccine-elicited murine antibodies were synthesized and cloned (GenScript) into pVRC8400 mammalian expression vectors encoding species-specific heavy- and light-chain constant regions, respectively. Immunoglobulin G (IgG) for ELISA and neutralization assays was expressed in Expi293F cells and purified using standard methods that were previously described^49^. To obtain D48 Fab for SPR and cryo-EM studies, a HRV 3C protease cleavage site (LEVLFQGP) was inserted into the hinge region of the plasmid encoding the full-length D48 IgG heavy chain. D48 bearing the HRV 3C cleavage site was expressed and purified as described above by swapping in the modified heavy-chain plasmid to be paired with the plasmid encoding the natural light chain. Then, 2 mg D48 IgG with the HRV 3C cleavage site was digested with HRV 3C protease (Thermo Fisher Scientific) for ~20 h at 4° C, from which liberated Fab fragments were purified using negative selection on a protein A column. To obtain WS.HSV-1.24 Fab for the SPR and cryo-EM studies, purified IgG was first reduced and alkylated using previously described methods^50^, followed by digestion with immobilized papain (Thermo Fisher Scientific) at 37 °C for 18 h in 1× phosphate-buffered saline (PBS), 120 mM NaCl, 30 mM cysteine and 1 mM EDTA (pH 7). To obtain Fabs of the antibodies DL16, SS10 and SS55 for SPR analysis, purified IgG was digested with immobilized papain (Thermo Fisher Scientific) at 37 °C for 5 h in 1× PBS, 120 mM NaCl, 30 mM cysteine and 1 mM EDTA (pH 7). All Fabs were then purified after papain digestion using negative selection on a protein G column by gravity flow and buffer exchanged by gel filtration using a Superdex 75 Increase 10/300 column. To obtain the HSV-1 gB co-receptor^32^ for SPR analysis, the ectodomain of PILRα was obtained from Addgene (plasmid 157142) and cloned into a pVRC8400 mammalian expression vector with an N-terminal single-chain Fc tag (scFc) and a HRV 3C cleavage site linker using In-Fusion Cloning (Takara Bio). An R78G substitution, which has been shown to increase the affinity of PILRα for gB^51^, was introduced by site-directed mutagenesis using KOD Xtreme Hot Start DNA Polymerase (Sigma–Aldrich). The scFc-PILRα_G78 construct was expressed and purified similarly to the antibodies described above. That is, 2 mg scFc-PILRα_G78 was digested with HRV 3C protease (Thermo Fisher Scientific) for ~20 h at 4 C, from which the liberated PILRα_G78 ectodomain fragment was purified using negative selection on a protein A column by gravity flow.

### Negative-stain electron microscopy

Carbon-coated 200 mesh copper grids (Electron Microscopy Sciences) were glow discharged using a PELCO easiGlow before the addition of 3 µl purified HSV-1 gB constructs diluted to 0.01–0.05 mg ml^−1^ in 10 mM HEPES and 150 mM NaCl (pH 7.5) buffer. After 45 s, protein samples were removed and grids were washed three times with 10 µl buffer, then negatively stained with 10 µl 1% uranyl acetate for 45 s. Micrographs were collected on an Hitachi HT7800 electron microscope operated at 100 kV and equipped with a TVIPS F416 CMOS 4k × 4k detector at a nominal magnification of 57,000, corresponding to a pixel size of 0.2 nm. Particle picking and two-dimensional (2D) classification were performed using cryoSPARC^52^.

### Single-particle cryo-EM

The high-resolution unbound (apo) and neutralizing antibody-bound structures of each gB-Ecto construct were determined using single-particle cryo-EM. Copper C-flat Holey carbon-coated grids (CF-1.2/1.3; 300 mesh; Electron Microscopy Sciences) were glow discharged using a PELCO easiGlow before the addition of 3 µl sample with 0.005% (wt/vol) *n*-dodecyl β-D-maltoside detergent and a final gB concentration of 2.0–2.5 mg ml^−1^. To prepare WS.HSV-1.24 and D48-bound complexes, each gB-Ecto construct was mixed with Fab at a 1:3 molar ratio and incubated on ice for 20 min. Samples were vitrified in liquid ethane using a Vitrobot Mark IV with a wait time of 30 s and a blot time of 4 s at room temperature with 100% humidity.

Cryo-EM data were collected on an FEI Titan Krios electron microscope operating at 300 kV, equipped with a Gatan K3 direct detector operating in counting mode using Leginon^53^. A total exposure dose of 58 *e*^−^ Å^−^^2^ was fractionated over 50 raw frames and a defocus range of 0.8–2.0 µm was used. All data processing—including motion correction, contrast transfer function estimation, particle picking and extraction, 2D classification, ab initio model generation and 3D refinements—was performed using cryoSPARC^52^. Non-templated particle picking was performed twice for each sample, to identify both pre- and postfusion particles without bias: once using a circular blob (100–280 Å) for the prefusion conformation; and once using an eclipse blob (100–340 Å) for the postfusion conformation. All homogenous 3D refinements were performed with C1 symmetry and all non-uniform 3D refinements were performed with C3 symmetry. 2D class averages and 3D reconstructions for all gB-Ecto constructs (both apo and antibody bound) each showed disordered low-resolution density emanating from regions of the gB trimer corresponding to the fusion loops at the tip of DI. We propose that this density corresponds to a second trimer that is forming an asymmetrical dimer of trimers, similar to our observations by negative-stain electron microscopy. In fact, at least one 2D class average for each apo and antibody-bound gB-Ecto construct stabilized in the prefusion conformation was a clear dimer of trimers. However, particle picking with larger diameters and extracting particles with larger box sizes had marginal effects on improving the 3D reconstruction density of the second trimer; therefore, refinements focused on just a single trimer.

All atomic models were solved with iterative rounds of manual model rebuilding in Coot^54^ and automated real-space refinement of the model in Phenix^55^. For atomic building of apo prefusion gB-Ecto constructs, we first used the pseudoatomic-level cryo-electron tomography structure (PDB ID: 6Z9M) as the initial model to fit into our 3.0-Å cryo-EM reconstruction of gB-Ecto.H516P.L531E.DS using UCSF ChimeraX^56^. After refinement, the structure of gB-Ecto.H516P.L531E.DS served as the initial model to fit into our 3.7-Å cryo-EM reconstruction of gB-Ecto.H516P.L531E. For atomic building of the apo postfusion gB-Ecto construct, we used the original gB ectodomain crystal structure (PDB ID: 2GUM)^21^ as the initial model to fit into our 2.9-Å cryo-EM reconstruction of gB-Ecto.H516P. For atomic building of the WS.HSV-1.24–gB and D48–gB complexes, the initial models for the WS.HSV-1.24 Fab and D48 Fab variable regions (Fv) were first obtained using the ABodyBuilder2 application of the SAbPred Antibody Prediction Toolbox^57^. For each complex, the respective Fv models and our solved apo pr- or postfusion gB-Ecto structures were fit into each respective cryo-EM 3D reconstruction density using UCSF ChimeraX^56^. The overall structure quality for all atomic models determined here was periodically assessed using MolProbity^58^ and EMRinger^59^ during refinement until satisfactory validation of the model was achieved. Summaries of cryo-EM data collection, 3D reconstruction and model refinement statistics for each structure are provided in Supplementary Figs. 1, 7 and 8, and Supplementary Tables 7–9.

### Calculations of domain movements

From the PDB pre- and postfusion conformations, the centroid of each domain was calculated using Cα atoms, then the centroid for the domain with least change in position between pre- and postfusion conformations was chosen as a reference and a reference rotation matrix was calculated. Relative rotations and translations were calculated for pre- and postfusion conformations of each domain, with output in Euler angles and degrees.

### SPR analysis

SPR binding assays were performed using a Biacore T200 biosensor equipped with a Series S Ni-NTA sensor chip in a running buffer of 10 mM HEPES (pH 7.4), 150 mM NaCl, 0.1 mg ml^−1^ bovine serum albumin and 0.005% (vol/vol) Tween 20 at 25 °C. For each binding cycle, Ni-NTA surfaces were charged using 0.5 mM NiSO_4_, and three different glycoproteins (gB-Ecto.H516P.L531E.DS, gB-Ecto.H516P.L531E and gB-Ecto.H516P) were captured through their C-terminal His-tag on independent flow cells at 250–350 response units (RU). An empty flow cell served as a reference flow cell for the subtraction of refractive index shifts. Fabs SS10, SS55, DL16 and WS.HSV-1.24 were diluted in the running buffer using a threefold dilution series ranging from 3.33–270 nM. D48 was tested at a dilution series ranging from 1.11–90.00 nM, whereas PILRα_G78 was tested at concentrations ranging from 10–810 nM to account for its lower affinity with each glycoprotein. Protein samples were tested at increasing concentrations and each series was tested in duplicate. For each binding cycle, the association phase over the four flow cells was monitored for 120 s at 50 µl min^−1^, followed by a dissociation time of 600 s for D48 and SS10, 300 s for SS55, 120 s for DL16 and 60 s for PILRα_G78. The Ni-NTA surfaces were regenerated using two consecutive pulses of 350 mM EDTA, followed by a buffer wash. Buffer injections were performed instead of protein samples every two cycles to remove systematic noise and drift from the binding signal. The data were processed and fit to a 1:1 interaction model using Scrubber 2.0 (BioLogic Software). For each set of kinetic parameters reported, the number in brackets represents the error of the fit. For the peptide24 binding experiment, WS.HSV-1.24 IgG was tethered to the sensor chip surface and binding of the peptide (DWVPKRPSVC) and its KLH conjugate, each at 120 nM, respectively, was tested for 300 s at 20 µl min^−1^.

### Murine immunizations

Groups of ten C57BL/6 mice (five males and five females per group) were immunized with gB-Ecto.H516P (postfusion) or gB-Ecto.H516P.L531E.DS (prefusion) twice at weeks 0 and 3. One group of ten BALB/c mice (five males and five females) was immunized with gB-Ecto.H516P.L531E.DS at weeks 0 and 3. All mice were obtained from The Jackson Laboratory and were approximately 6 weeks old when used for this study. All immunizations comprised 10 µg gB-Ecto protein adjuvanted with 50 µg poly(I:C) (Sigma–Aldrich), administered intramuscularly into the caudal thighs via needle syringe. Terminal blood draws were taken at week 5 and sera were isolated once blood draws had coagulated. Sera samples were assessed by ELISA and live-virus neutralization, with data collection and analyses performed blind to each experimental group. No statistical methods were used to pre-determine sample sizes, but our sample sizes are similar to those reported in previous publications^3^. Mice were housed in ventilated cages (with a maximum of five mice per cage) under 12-h light and 12-h dark cycles with controlled temperature and humidity.

### Murine hybridoma generation and antibody isolation

Two prefusion gB-immunized BALB/c mice (L03 and L05) were selected for a third boost of adjuvanted gB-Ecto.H516P.L531E.DS at week 5 rather than undergoing terminal blood draws with the remainder of the group. Three days later, splenocytes were harvested and hybridomas were generated by fusing with Sp2/0 myeloma cells (CRL-1581; ATCC) using standard electrofusion parameters and hypoxanthine-aminopterin-thymidine medium selection^60^. After 9 d culture, hybridoma well supernatants were harvested and screened by ELISA for recognition of gB-Ecto.H516P (postfusion) or gB-Ecto.H516P.L531E (prefusion) using the ELISA methods described above. IgG was purified from the supernatants of select prefusion-specific hybridoma culture wells using protein G (Thermo Fisher Scientific) and tested by ELISA for pre- and postfusion gB recognition to confirm conformational specificity. Heavy and light chain variable regions were sequenced from validated prefusion-specific culture wells by generating complementary DNA libraries and using two rounds of nested PCR to amplify immunoglobulin genes^61^. Sequences were analysed using IMGT/V-QUEST to identify germline immunoglobulin genes and quantify levels of somatic hypermutation^62,63^.

### HSV-1 neutralization assay

An end-point dilution microplate assay was used to measure the neutralization activity of monoclonal antibodies and animal serum samples. In brief, serum samples were heat inactivated and subjected to serial fivefold dilutions starting from 1:50, whereas antibodies were subjected to serial dilutions starting at 50 µg ml^−1^. Triplicates of each dilution were incubated with HSV-1 MacIntrye (VR-539; ATCC) authentic live virus at a multiplicity of infection of 0.1 in EMEM with 5% heat-inactivated foetal bovine serum for 1 h at 37 °C. After incubation, the virus–serum or virus–antibody mixtures were transferred onto a monolayer of Vero cells (CRL-1586; ATCC) with a density of 4 × 10^4^ per well grown overnight. The cells were incubated with the mixture for 48 h. The cytopathic effect of viral infection was visually scored for each well in a blinded fashion by two independent observers. The results were then reported as a percentage of neutralization at a given sample dilution compared with virus-only control wells. After subtracting the cell-only control groups, the 50% inhibitory dose (ID_50_) and 50% inhibitory concentration (IC_50_) values of each sample were calculated using five-parameter dose-response nonlinear regression in GraphPad Prism. Live-virus propagation and neutralization assays were performed within a Biosafety Level 2 facility according to the laboratory safety guidelines from the Institutional Biosafety Committee at Columbia University (Appendix #BJFZ7509).

### HSV-1 gB ELISAs

Titres for the binding of polyclonal serum IgG and monoclonal IgG to pre- and postfusion gB were determined by ELISA. High-binding 96-well plates (Corning Costar) were coated with 1 µg ml^−^^1^ gB-Ecto.H516P (postfusion) or gB-Ecto.H516P.L531E.DS (prefusion) diluted in 1× PBS (pH 7.4) and incubated overnight at 4 °C. Plates were then washed three times with PBS-T (1× PBS (pH 7.4) and 0.1% Tween 20 detergent) and then coated with blocking buffer (1× PBS, 20% heat-inactivated foetal bovine serum and 2% bovine serum albumin) and incubated for 1.5 h at 37 °C. Plates were then washed three times with PBS-T, coated with serial fivefold dilutions in blocking buffer of heat-inactivated sera starting from 1:100 or monoclonal antibodies starting from 5 or 10 µg ml^−1^ and incubated for 1 h at 37 °C. Plates were then washed three times with PBS-T and detected with HRP-goat anti-mouse IgG (115-035-008; The Jackson Laboratory) or HRP-goat anti-human IgG (109-035-088; The Jackson Laboratory) diluted to 1:5,000 or 1:10,000 in blocking buffer, respectively. After the plates were incubated for 1 h at 37 °C, they were washed three times with PBS-T, developed with Ultra TMB-ELISA substrate solution (Thermo Fisher Scientific) for 5 min at room temperature and then quenched with an equal volume of 0.2 M sulfuric acid. The absorbance was measured at 450 nm within 2 min of quenching. After subtracting the signal from serum-negative control wells, values for the dose (ED_50_) or concentration (EC_50_) of each sample required to produce 50% of its maximal effect were calculated using five-parameter dose-response nonlinear regression in GraphPad Prism. For the time course ELISAs, plates were coated with 1 µg ml^−1^ gB-Ecto.H516P (postfusion) or gB-Ecto.H516P.L531E.DS (prefusion) and incubated at 4 °C for the precise indicated times (1, 5 or 25 h) before washing and blocking as described above. The area under the curve for post- and prefusion gB at each timepoint was calculated using GraphPad Prism.

### Infectious HSV-1 virion ELISA

Titres for the binding of gB-reactive antibodies to infectious viral particles were determined by virion-binding ELISA. Briefly, HSV-1 viruses were propagated in Vero cells (CRL-1586; ATCC) and purified by ultracentrifugation with a 25% sucrose cushion at 22,000*g* and 4 °C. Infectious particle numbers were determined through a 50% tissue culture infectious dose titration. High-binding 96-well plates (Corning Costar) were coated with purified HSV-1 MacIntrye (VR-539; ATCC) authentic live virus at 50,000 infectious particles per well in 0.1 M NaHCO_3_ and incubated overnight at 4 °C. Plates were then washed three times with PBS-T (1× PBS (pH 7.4) and 0.5% Tween 20 detergent), then coated with blocking buffer (1× PBS, 5% milk and 0.5% bovine serum albumin) and incubated for 1 h at 37 °C. Plates were then washed three times with PBS-T, coated with serial fivefold dilutions in blocking buffer of monoclonal antibodies starting from 20 μg ml^−1^ and incubated for 1 h at 37 °C. Plates were then washed three times with PBS-T and detected with HRP-goat anti-mouse IgG (31430; Invitrogen) diluted to 1:5,000 in blocking buffer. After plates were incubated for 1 h at 37 °C, they were washed eight times with PBS-T, developed with 3,3′,5,5′-tetramethylbenzidine substrate (Sigma–Aldrich) for 15 min at room temperature and then quenched with an equal volume of 0.5 M sulfuric acid. The absorbance was measured at 450 nm within 2 min of quenching. Signals were subtracted from buffer-only control wells and plotted using GraphPad Prism.

### Surface and refolding analysis

Accessible surface areas were defined using the Python FreeSASA library, with a probe size of 5 Å, which is more in line with that of an amino acid, to better simulate recognition by antibodies^64^ rather than the typical 1.4 Å used to mimic water. Domain interfaces were defined as those residues residing at the N- and C-terminal regions of each domain, which were present in both pre- and postfusion structures but did not possess secondary structures. Epitopes were defined using solvent-accessible surface area calculations (with a probe size of 5 Å for access) and the Python FreeSASA library. Refolding analysis utilized a sliding window of 11 amino acids, as previously described^37^, to align and calculate backbone (Cα) RMSD values between pre- and postfusion gB using PyMOL (version 2.6). The refolding regions were defined as two or more consecutive residues with >2.5 Å RMSD.

### HDX analysis

H_2_O buffer (10 mM HEPES and 150 mM NaCl (pH 7.5)) was prepared in deionized water and lyophilized using Labconco FreeZone 4.5 lyophilizer for at least 24 h, then resuspended in D_2_O to produce deuterated buffer. The protein stock solution (3.5 mg ml^−1^ prefusion gB or 1.7 mg ml^−1^ postfusion gB) was diluted tenfold in 75 µl deuterated buffer and exchanged at 15 °C for the specified time, then diluted twofold in a quench solution (3% acetonitrile and 1% formic acid in H_2_O; all MS-grade; Thermo Fisher Scientific) at 2 °C. Then, 140 μl of the quenched reaction was injected into a refrigerated protease column. Solvent A (3% acetonitrile and 0.15% formic acid in H_2_O) was pumped (UltiMate 3000; Thermo Fisher Scientific) at 150 μl min^−1^ through the protease column. Peptides eluted from the protease column were routed to a 1 mm × 10 mm C18 trap column (Hypersil GOLD; 3-μM pore size; Thermo Fisher Scientific). Two technical replicates were performed on different days with different proteases and timepoints. For the first replicate, we used an immobilized fungal protease type XIII/pepsin column (FP, 1:1 wt/wt, 2.1 mm × 30 mm; NovaBioAssays). For the second replicate, we used an alanyl aminopeptidase/pepsin column (AP/pepsin 2.1 mm × 20 mm; Affipro). Hydrogen exchange liquid handling was performed manually for the first replicate and using the LEAP system (Trajan) controlled by Chronos 5.8.3 software (Trajan) for the second replicate.

After digesting the proteins to peptides and desalting, the system valve was switched so that the binary pump (Infinity II; Agilent) was in line with the C18 trap column, flowing in the opposite direction. The binary pump used a gradient of solvent A and solvent B (96.85% acetonitrile and 0.15% formic acid) to elute peptides from a 50 mm × 1 mm C18 analytical column (Hypersil GOLD; 1.9-μM pore size; Thermo Fisher Scientific). The elution method comprised isocratic flow at 10% B for 1 min, a linear gradient to 40% B from 1–15 min, a linear gradient to 95% B from 15–19 min, isocratic flow at 100% B from 19–24 min, a linear gradient to 10% B from 24–30 min and then isocratic flow at 10% B from 30–45 min, at a flow rate of 0.04 ml min^−1^. In the second replicate, the protease column was washed between sample injections with injections of quench solution, and an auxiliary pump (MX-Class; Teledyne Labs) flowed additional solvent A over the C18 trap at 150 μl min^−1^ during the desalting step.

Eluted peptides entered the mass spectrometer (Bruker maXis-II ETD) via electrospray ionization, where full-scan mass spectra were collected in positive ion mode from 100–2,250 *m/z* with a 1.0-Hz spectra rate, 500-V end-plate offset, 4,200-V capillary, 1.7-bar nebulizer, 8 l min^−1^ dry gas and 180 °C dry temperature. We also ran tandem mass spectrometry (MS/MS) experiments for each sample with the same full mass spectrometry settings as described above, but with a spectra rate of 1.20 Hz and a dry temperature of 200 °C. We used Bruker Compass HyStar 5.1 software to acquire the data.

We used Compass DataAnalysis 5.1 (Bruker) to identify and deconvolute peptides, and PIGEON for peptide matching and disambiguation^65^. Using the prefusion gB sample MS/MS dataset, we applied the following peptide identification settings: 30-ppm initial threshold; 7-ppm narrow threshold; 0.020-Da error for MS/MS; 0.05 score threshold; peptide length of 3–15; charge threshold of 3; and 0.1 Daltons per elementary charge (D/e) and 0.5-min degeneracy thresholds. The resultant peptide pool was imported to HDExaminer 3.3.0 (Sierra Analytics) and all spectra were fit for each replicate. All peptide assignments and fits were manually checked for quality. Peptide pool results and spectra were exported from HDExaminer and used for peptide-level analysis of both replicates in FEATHER^65^, with envelope centroids used to produce deuterium uptake plots. Deuterated isotopic mass envelope centroids were compared with undeuterated envelope centroids and the number of exchangeable residues in each peptide to determine deuteration levels as percentages of the maximum theoretical exchange levels. Deuteration levels of all peptides covering each residue were averaged and subtracted between the pre- and postfusion states, then averaged across all timepoints to calculate the pseudo-residue level $\Delta \bar{D}$. The full peptide and timepoint HDX and mass spectrometry data are available in Supplementary Fig. 3.

### Glycan shield analysis

The full glycan structure was added using GlycoSHIELD^66^, which models the Man5 glycan at experimentally validated *N*-linked glycan residues 141, 398, 430 and 674. The glycans were further refined using a coarse-grained method with default parameters, generating 30 frames of glycan conformations. The average number of glycan atoms per residue was calculated using GLYCO^63,67^ under a multiple-frame model, with the glycan cutoff set at 15 Å. To determine conservation of HSV-1 *N*-linked glycans among alphaherpesviruses, the following representative sequences were analysed: HSV-1 KOS isolate (E03113.1; GenBank); HSV-2 HG52 isolate (CAB06752.1; GenBank); pseudorabies virus Indiana-Funkhauser isolate (AAA47465.1; GenBank); and HHV-3 (varicella zoster virus) isolate VR1 (AGY33060.1; GenBank). A potential *N*-linked glycan site was considered to be conserved with HSV-1 if it fell within a three-residue span of HSV-1 residues 87, 141, 398, 430, 489 and 674 in a sequence alignment of alphaherpesvirus gBs.

### Cell-surface recognition of full-length HSV-1 gB

To express HSV-1 gB on the cell surface, Expi293F cells were transiently transfected with plasmid DNA (1 µg DNA per ml culture) encoding full-length wild-type HSV-1 KOS gB or HSV-1 KOS gB with stabilization of the prefusion conformation (gB-Ecto.H516P.L531E) using PEI MAX at a 1:3 ratio (DNA:PEI). Two days after transfection, cells were harvested by centrifugation, washed twice with PBS-F (1× PBS + 5% foetal calf serum) and stained with LIVE/DEAD Violet (Thermo Fisher Scientific) for 20 min at room temperature. Cells were then washed twice with PBS-F and incubated with 25 µg ml^−1^ mouse or human antibodies for 20 min at room temperature. Cells were then washed twice with PBS-F and stained with Alexa Flour 647-conjugated anti-mouse IgG (115-605-003; The Jackson Laboratory) or Alexa Flour 488-conjugated anti-human IgG (109-545-008; The Jackson Laboratory) diluted 1:200 in staining buffer (1× PBS + 5% foetal calf serum and 0.1% sodium azide) for 30 min on ice. Cells were then washed twice with staining buffer and analysed by fluorescence-activated cell sorting using an LSRFortessa (BD Biosciences). Flow cytometry data were analysed using FlowJo version 10 software.

### Statistics and reproducibility

Differences between binding and serum neutralization titres were determined using a two-tailed Student’s *t*-test and GraphPad Prism. Data distributions were assumed to be normal, but this was not formally tested. The investigators were blinded to experimental group allocation during the experiments and data analyses.

### Reporting summary

Further information on research design is available in the Nature Portfolio Reporting Summary linked to this article.

## Supplementary information

Supplementary Information Supplementary Figs. 1–8 and Tables 1–9.

Reporting Summary

## Source data

Source Data Figs. 2–5 and Extended Data Figs. 3, 4, 6 and 7 Source numerical data.

## Data Availability

Coordinates for the apo gB-Ecto cryo-EM structures have been deposited in the Protein Data Bank (PDB) with the accession codes 9DDA, 9DDB and 9DDC, and the corresponding cryo-EM 3D reconstruction maps have been deposited in the Electron Microscopy Data Bank (EMDB) with the accession codes 46762, 46763 and 46765, respectively. The cryo-EM structure coordinates for the D48-bound gB-Ecto complexes have been deposited in the PDB with the accession codes 9DD7, 9DD8 and 9DD9, and the corresponding cryo-EM 3D reconstruction maps have been deposited in the EMDB with the accession codes 46759, 46760 and 46761, respectively. The cryo-EM structure coordinates for the WS.HSV-1.24-bound gB-Ecto.H516P.L531E complex have been deposited in the PDB with the accession code 9DD6, and the corresponding cryo-EM 3D reconstruction map has been deposited in the EMDB with the accession code 46758. The HDX/MS dataset has been deposited on Zenodo (10.5281/zenodo.17354096)^68^. Source data are provided with this paper.

## Code availability

The solvent-accessible surface area Python scripts used for surface accessibility analysis of gB and other viral fusion machines were from the Python FreeSASA Library^69^ and can be accessed on GitHub (https://github.com/mittinatten/freesasa).
