# Supplementary material for: Prefusion structure, evasion and neutralization of HSV-1 glycoprotein B
Source: Nat Microbiol. 2025 Oct 31;10(11):2966–80. doi: 10.1038/s41564-025-02153-x (PMC12578645; doi:10.1038/s41564-025-02153-x)
Supplement: Supplementary file 1 — Supplementary Figs. 1–8 and Tables 1–9. [file 41564_2025_2153_MOESM1_ESM.pdf]

nature microbiology

Supplementary information

https://doi.org/10.1038/s41564-025-02153-x

Prefusion structure, evasion and neutralization of HSV-1 glycoprotein B

In the format provided by the authors and unedited

Supplementary Information

Supplementary Fig. 1-CryoEM validation apo gB

Supplementary Fig. 2-HDX/MS experiments and length-normalized analysis

Supplementary Fig. 3-HDX/MS analysis by timepoint

Supplementary Fig. 4-Bimodal HDX gB peptides

Supplementary Fig. 5-SPR binding kinetics for vaccine-elicited antibodies to three forms of gB ectodomain

Supplementary Fig. 6-FACS gating strategy for full-length gB recognition

Supplementary Fig. 7-CryoEM validation of gB-WS.HSV-1.24

Supplementary Fig. 8-CryoEM validation of gB-D48

Supplementary Table 1-Expession and conformational assessment of prefusion-stabilized gB designs

Supplementary Table 2-Prefusion and postfusion gB domain features and quantification of conformational rearrangements

Supplementary Table 3-HDX/MS experiments summary

Supplementary Table 4-Immunogenetics of vaccine-elicited prefusion gB-specific monoclonal antibodies isolated from mice

Supplementary Table 5-SPR affinity measurement values for vaccine-elicited antibodies to three forms of gB ectodomain

Supplementary Table 6-Quantified surface areas for the prefusion and postfusion conformations of diverse class III fusion machines.

Supplementary Table 7-Cryo-EM data collection, refinement and validation statistics for apo structures

Supplementary Table 8-Cryo-EM data collection, refinement and validation statistics for D48 complex structures

Supplementary Table 9-Cryo-EM data collection, refinement and validation statistics for the WS.HSV-1.24 complex structure

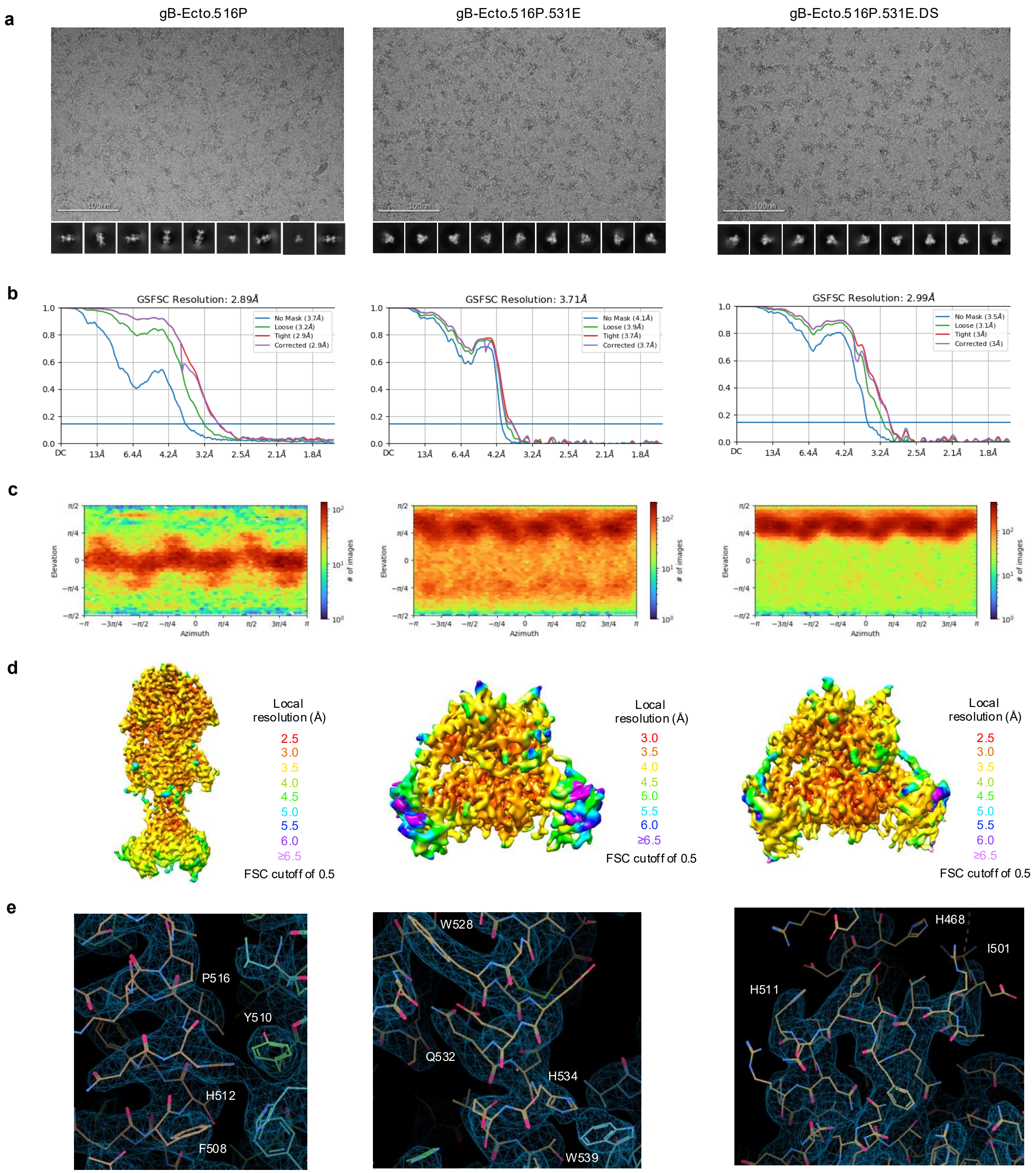

Supplemental Fig. 1 | Single-particle cryo-EM validation for apo HSV-1 gB ectodomains.
a, Representative raw micrograph with representative 2D class averages of picked particles shown below. **b**, Orientations of all particles used in the final refinement are shown as a heatmap. **c**, Gold-standard fourier shell correlation (FSC) curves with auto-tightening using a non-uniform refinement with C3 symmetry. **d**, Local resolution estimation of the full map is shown as generated through cryoSPARC using an FSC cutoff of 0.5. **e**, Example of high-resolution cryo-EM 3D reconstruction density.

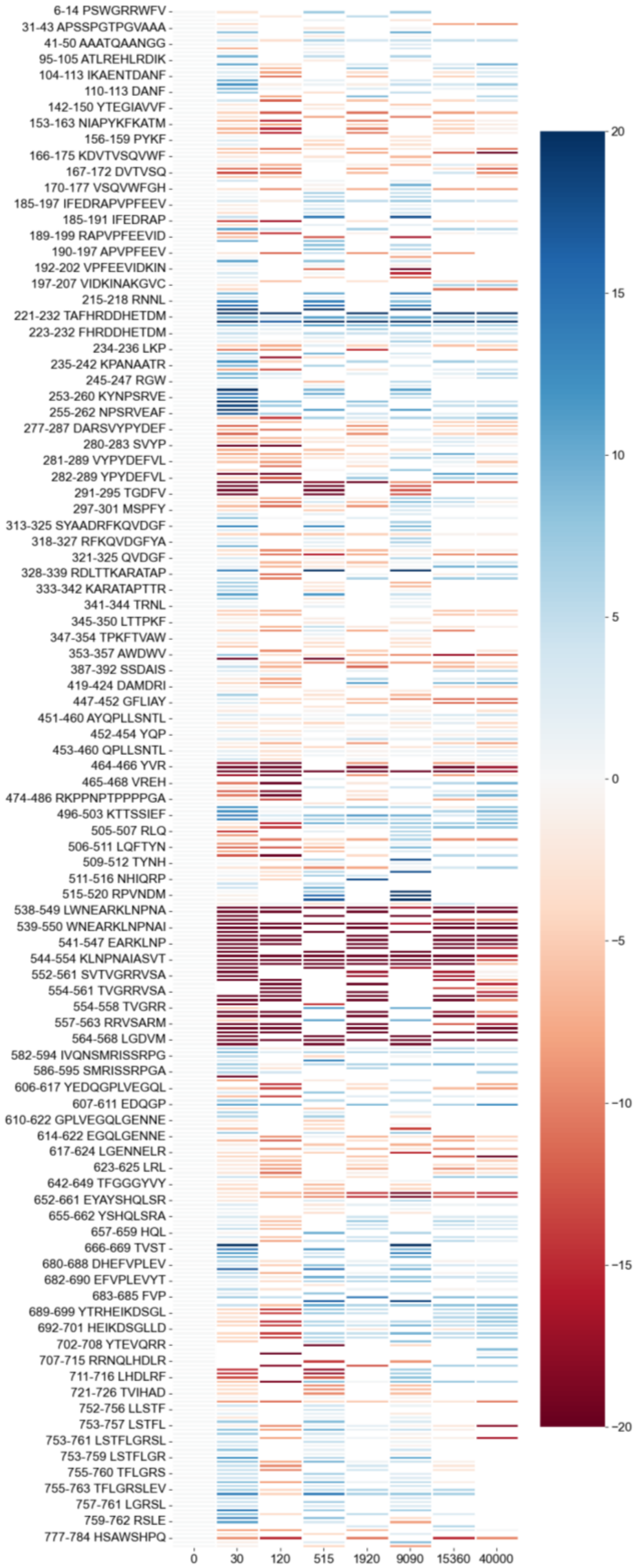

Supplemental Fig. 2 | HDX/MS analysis. Peptide-level length-normalized ΔDeuteration vs. time: prefusion gB - postfusion gB.

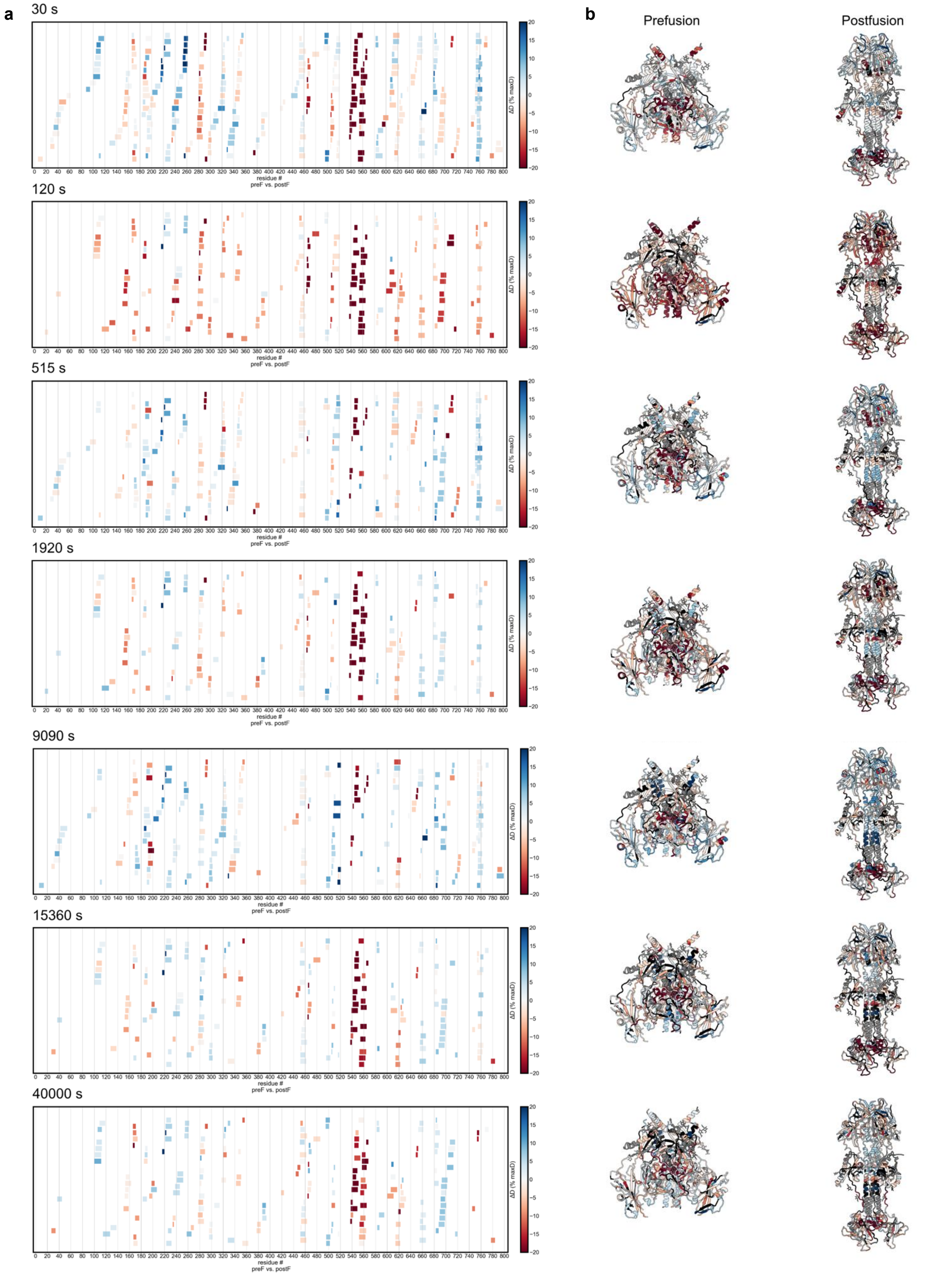

Supplemental Fig. 3 | HDX/MS analysis by timepoint. a, Single timepoint Woods plots. Red: more protected in prefusion state. **b,** Single timepoint structural representations of HX data. Red: more protected in prefusion gB.

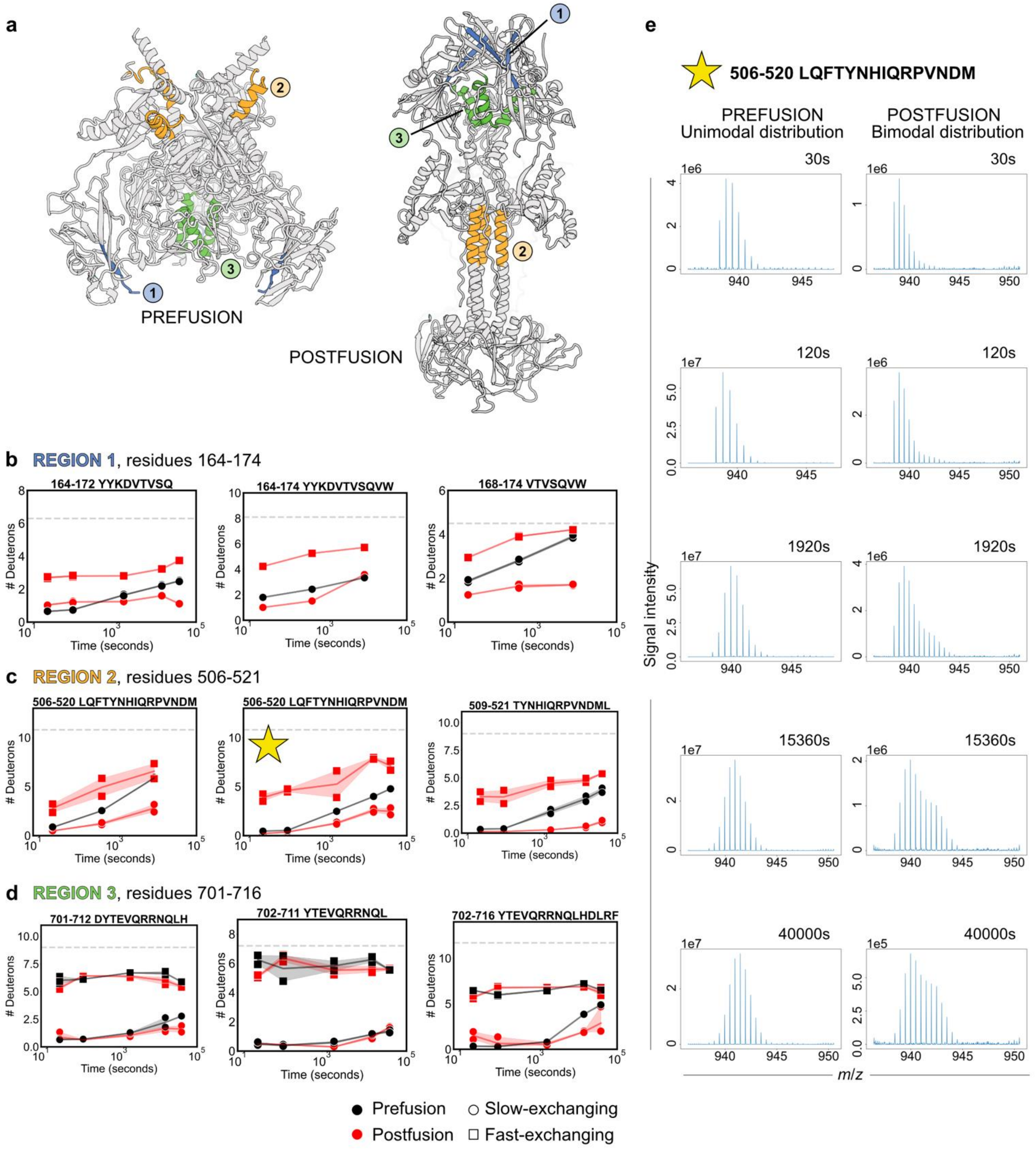

Supplemental Fig. 4 | Bimodal HDX gB peptides.
a, Regions of the HSV-1 gB protein that have bimodal peptide behavior. Regions 1 and 2 are bimodal only in postfusion gB. Region 3 is bimodal in both pre- and postfusion gB. **b-d**, Deuterium uptake plots corresponding to bimodal regions with representative uptake plots for each population. **e**, Full isotopic mass envelopes for the starred peptide in **c** (region 2) demonstrating bimodal behavior over time.

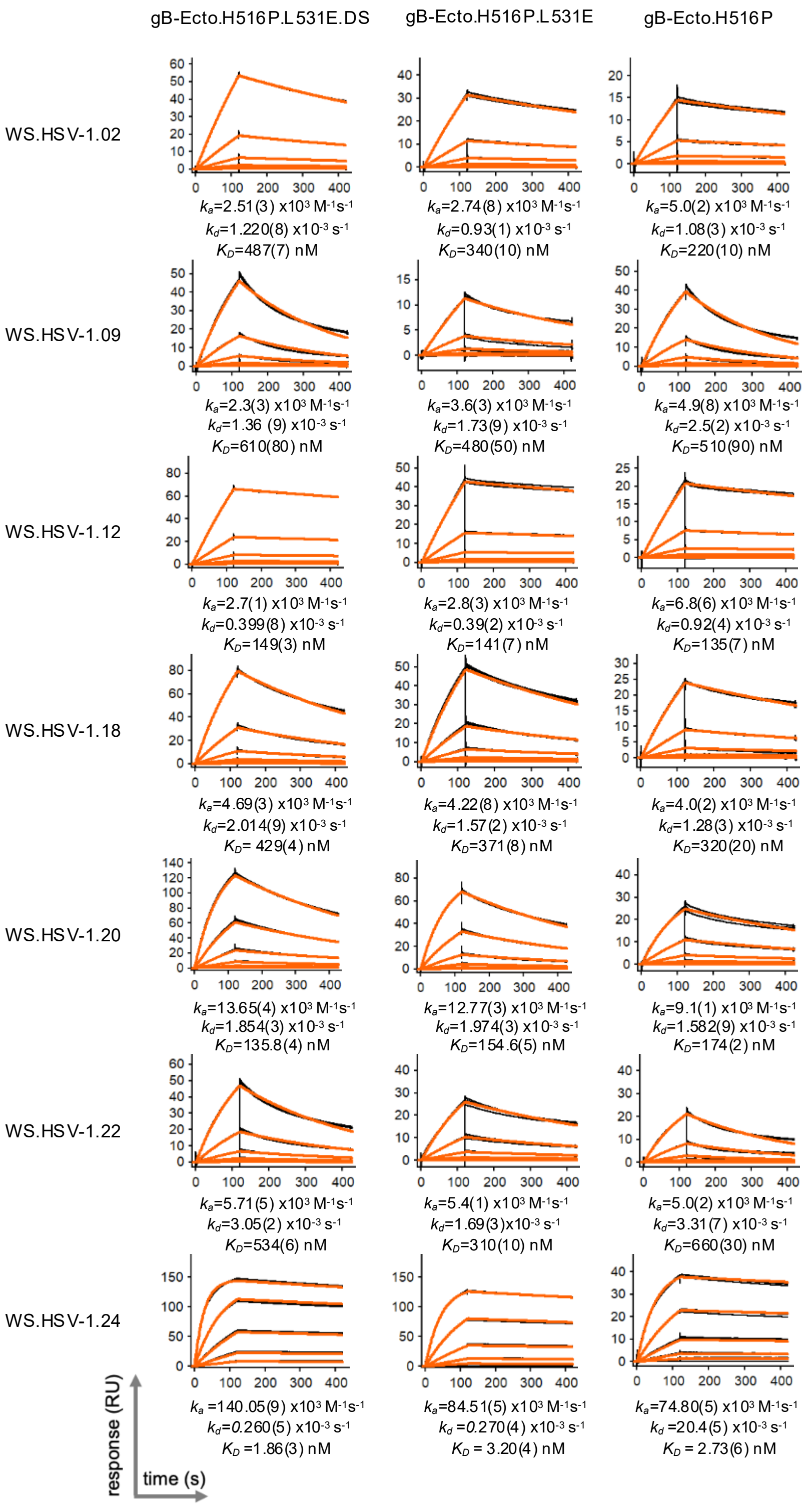

Supplemental Fig. 5 | Affinity measurements for vaccine-elicited antibodies to three forms of gB by SPR.
SPR kinetic binding assays with vaccine-elicited monoclonal antibodies. Whereas gB-Ecto.H516P was not recognized by ELISA, these antibodies can recognize and induce conformational changeof gB-Ecto.H516P in the soluble context of SPR.

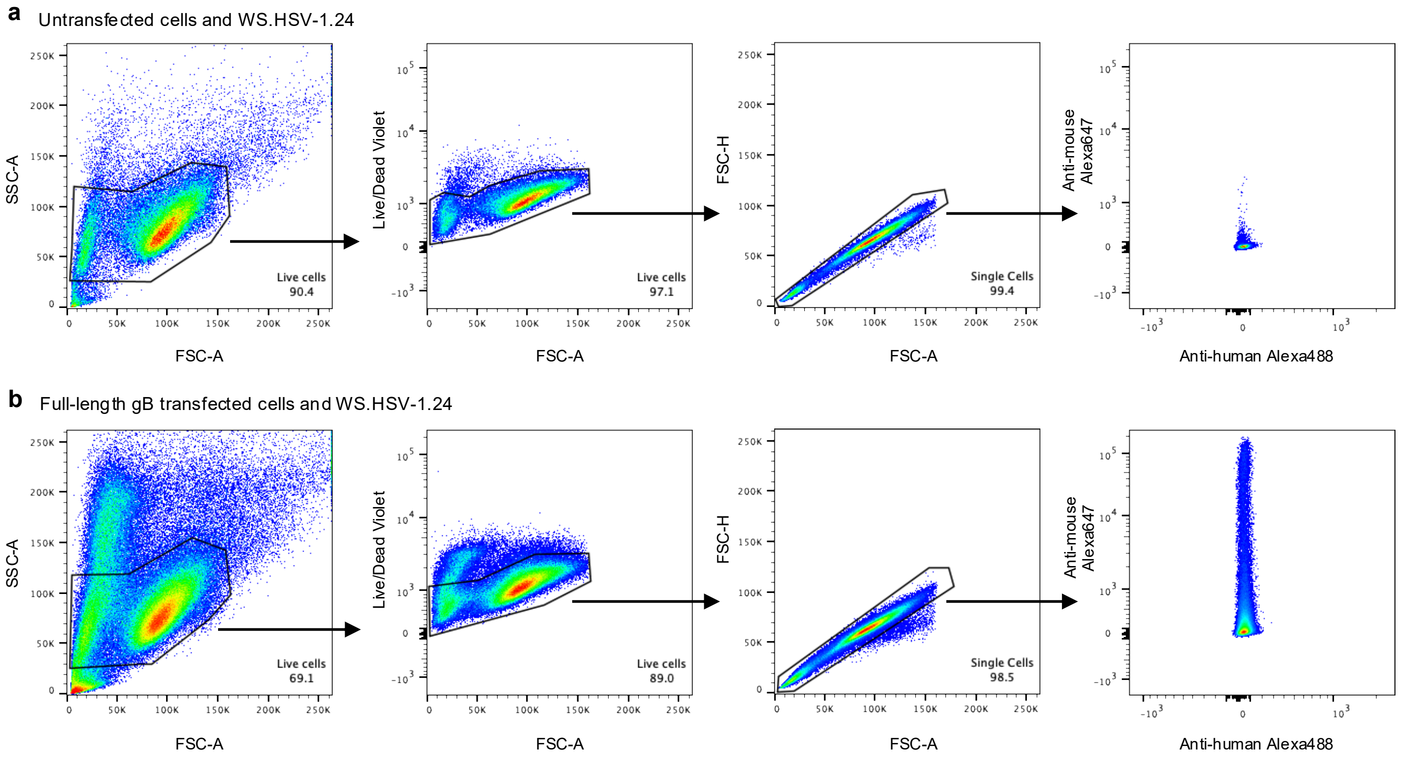

Supplemental Fig. 6 | FACS gating strategy for cell-surface recognition of full-length, membrane-bound gB.
a-b, Gating strategy for FACS experiments to analyze recognition of full-length gB. Representative data is provided for staining with vaccine-elicited murine antibody WS.HSV-1.24 on untransfected (a) and gB-transfected (b) cells.

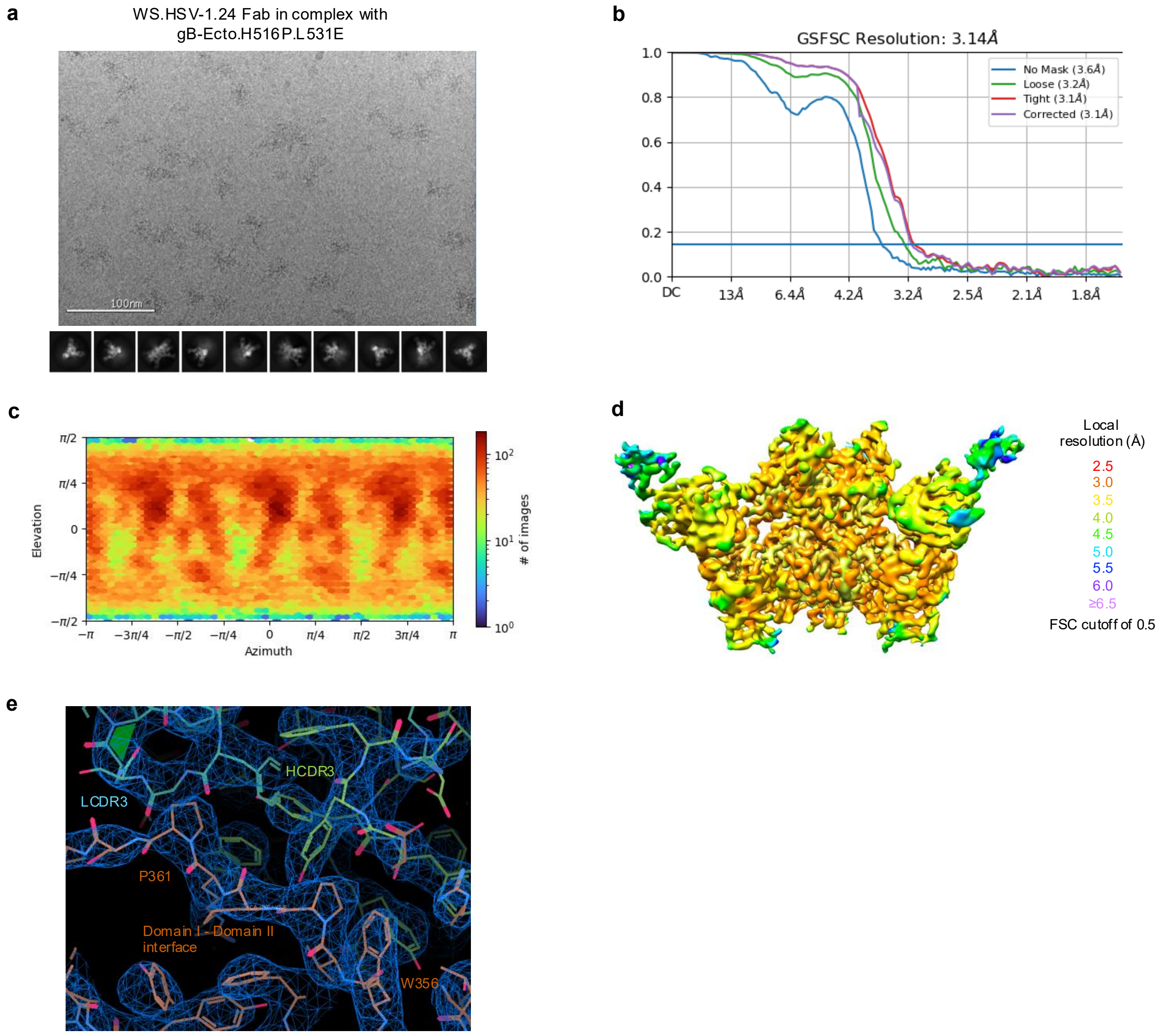

Supplemental Fig. 7 | Single-particle cryo-EM validation for HSV-1 gB in complex with WS.HSV-1.24.
a, Representative raw micrograph with representative 2D class averages of picked particles shown below. **b**, Orientations of all particles used in the final refinement are shown as a heatmap. **c**, Gold-standard fourier shell correlation (FSC) curves with auto-tightening using a non-uniform refinement with C3 symmetry. **d**, Local resolution estimation of the full map is shown as generated through cryoSPARC using an FSC cutoff of 0.5. **e**, Cryo-EM 3D reconstruction density to highlight the interactive surface with gB.

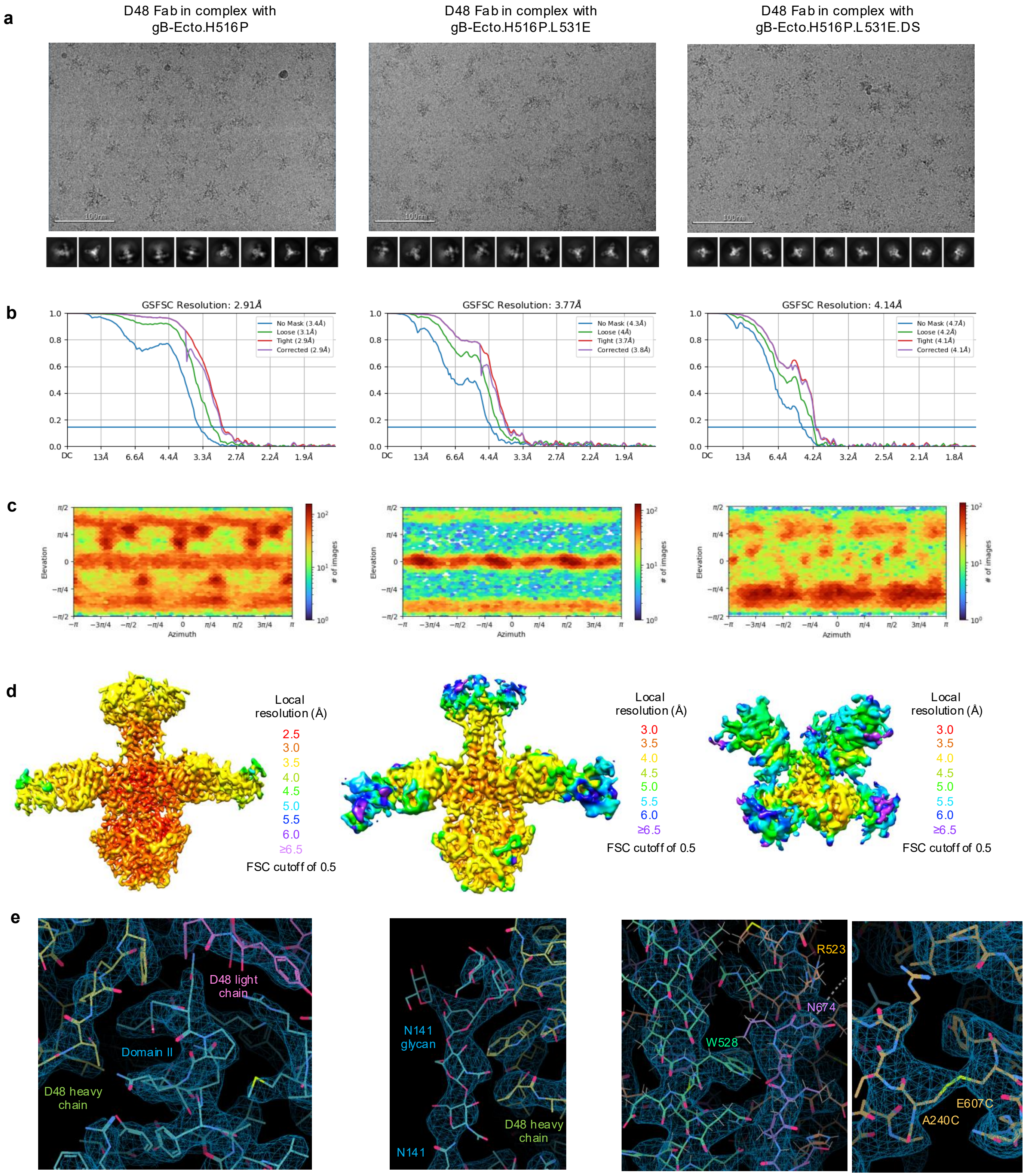

Supplemental Fig. 8 | Single-particle cryo-EM validation for HSV-1 gB complexes with D48.
a, Representative raw micrograph with representative 2D class averages of picked particles shown below. **b**, Orientations of all particles used in the final refinement are shown as a heatmap. **c**, Gold-standard fourier shell correlation (FSC) curves with auto-tightening using a non-uniform refinement with C3 symmetry. **d**, Local resolution estimation of the full map is shown as generated through cryoSPARC using an FSC cutoff of 0.5. **e**, Cryo-EM 3D reconstruction density to highlight features of structures.

Supplementary Table 1. HSV-1 gB ectodomain construct design, expression, and conformation.

Ectodomain construct	Stabilization strategy	Relative Expression	gB conformation
gB-Ecto.H516P with T4 foldon (base construct)	Disrupt extended postF DIII helix	+	Postfusion
gB-Ecto.H516P.V517E	Disrupt postF DIII helix bundle	--	n/a
gB-Ecto.H516P.L521E		--	n/a
gB-Ecto.H516P.V524E		--	n/a
gB-Ecto.H516P.W528E		--	n/a
**gB-Ecto.H516P.L531E**		++++	Prefusion
gB-Ecto.H516P.N238C+Q609C	Lock DI with interdomain disulfide bond	--	n/a
gB-Ecto.H516P.N238C+G610C		--	n/a
gB-Ecto.H516P.N238C+P611C		--	n/a
**gB-Ecto.H516P.A240C+E607C (DS)**		+	Prefusion with minor Postfusion fraction
gB-Ecto.H516P.T242C+Y606C		--	n/a
gB-Ecto.H516P.A290C+E679C		--	n/a
gB-Ecto.H516P.A290C+D680C		--	n/a
gB-Ecto.H516P.T291C+H681C		--	n/a
gB-Ecto.H516P.G292C+H681C		--	n/a
**gB-Ecto.H516P.L531E. A240C+E607C (DS)**	Combine Prefusion stabilizing substitutions	+++	Prefusion

Expression is relative to the base construct
Dashes -- indicate no expression
Conformation assessed by negative stain EM

Supplementary Table 2. Prefusion to postfusion transition of HSV-1 gB by domain.

Domain	PreF residues	PostF residues	RMSD (Ca; Å)	PreF-> PostF rotation X (°)	PreF-> PostF rotation Y (°)	PreF-> PostF rotation Z (°)	PreF-> PostF translation (Å)	*res Δdist > 2.5 Å (%)
I	157-359 (disordered 174-184, 252-266)	157-359	0.94	-163.8	47.33	-141.8	128.3	11.86
II	140-153, 364-473	140-153, 364-473	0.64	-173.4	-7.793	2.379	43.23	19.53
III	116-133, 510-573	116-133, 501-573	2.32	-60.84	72.64	1.779	6.176	38.46
IV	111-115, 574-664	111-115, 574-664	0.45	27.56	40.52	-165.2	0.03237	0.0000
V	670-721 (disordered 722-725)	670-725	13.72	-147.4	41.17	-108.4	89.39	69.23

*Percent of domain residues with pairwise C-alpha distance over 2.5 Å after superimposition

Supplementary Table 3. HDX/MS experiments summary.

Dataset	**HSV-1 gB ectodomains, Run 1**	**HSV-1 gB ectodomains, Run 2**
Protein states	Prefusion H516P.L531E /Postfusion H516P	Prefusion H516P.L531E /Postfusion H516P
Date	1/8/2025	1/22/2025
Protease column	Fungal protease XIII/ pepsin	ANPEP/pepsin
Reaction details	10mM HEPES, 150mM NaCl, pH 7.5, temperature 15 °C, final D_2O concentration 90%	
Time course (s)	0, 30, 515, 9090	0, 30, 120, 1920, 15360, 40000
# of time points	3	5
# of peptides	416	316
Sequence coverage	88.5%	85.8%
Average peptide length / Redundancy	9.7 length, 5.0 redundancy	10.1 length, 3.9 redundancy

Supplementary Table 4. Immunogenetics of selected prefusion gB-specific monoclonal antibodies isolated from hybridoma culture screening.

Mouse	mAb ID	IGVH	%nt VH SHM	IGJH	IGDH	VDJ Junction	IGVK	%nt VK SHM	IGJK	VJ Junction
L03	WS-HSV-1.02	IGHV1-7*02	3.1	IGHJ4*01	IGHD2-14*01	ARGDYRYHYYAMDY	IGKV3-1*01	0.0	IGKJ1*01	QQSRKVPWT
L03	WS-HSV-1.09	IGHV1-47-6*01	5.6	IGHJ4*01	IGHD2-14*01	ARRGYDGALDY	IGKV6-17*01	3.2	IGKJ1*01	QQHYSTPWT
L03	WS-HSV-1.12	IGHV1-7*02	2.4	IGHJ4*01	IGHD2-14*01	ARGDYRYHYFAMDS	IGKV3-1*01	1.0	IGKJ1*01	QQSRKVPWT
L05	WS-HSV-1.18	IGHV8-4-19*01	5.8	IGHJ4*01	IGHD1-1*01	VRRNNFGAMDY	IGKV12-41*01	1.4	IGKJ2*01	QHFWSSPYT
L05	WS-HSV-1.20	IGHV8-4-4*01	2.4	IGKJ1*01	IGHD1-1*01	ARISSWYFDV	IGKV12-41*01	0.7	IGKJ2*01	QHFWSIPYT
L05	WS-HSV-1.22	IGHV1-47-2*01	2.4	IGKJ1*01	IGHD1-1*01	TRRAVVPAWYFDV	IGKV6-15*01	1.8	IGKJ2*01	QQYNNYPYT
L05	WS-HSV-1.24	IGHV2-3*01	2.5	IGKJ2*01	IGHD1-1*01	CVSYYGYFDH	IGKV9-120*02	0.7	IGKJ1*01	LQYASSPPT

Supplementary Table 5. Affinity of prefusion-specific antibodies for the three forms of gB ectodomain measured by SPR.

	gB-Ecto.H516P.L531E.DS			gB-Ecto.H516P.L531E			gB-Ecto.H516P		
	k_a x 10^3 ($M^{-1}s^{-1}$)	k_d x 10^{-3} (s^{-1})	K_D (nM)	k_a x 10^3 ($M^{-1}s^{-1}$)	k_d x 10^{-3} (s^{-1})	K_D (nM)	k_a x 10^3 ($M^{-1}s^{-1}$)	k_d x 10^{-3} (s^{-1})	K_D (nM)
WS.HSV-1.02	2.51(3)	1.220(8)	487(7)	2.74(8)	0.93(1)	340(10)	5.0(2)	1.08(3)	220(10)
WS.HSV-1.09	2.3(3)	1.36(9)	610(80)	3.6(3)	1.73(9)	480(50)	4.9(8)	2.5(2)	510(90)
WS.HSV-1.12	2.7(1)	0.399(8)	149(3)	2.8(3)	0.39(2)	141(7)	6.8(6)	0.92(4)	135(7)
WS.HSV-1.18	4.69(3)	2.014(9)	429(4)	4.22(8)	1.57(2)	371(8)	4.0(2)	1.28(3)	320(20)
WS.HSV-1.20	13.65(4)	1.854(3)	135.8(4)	12.77(3)	1.974(3)	154.6(5)	9.1(1)	1.582(9)	174(2)
WS.HSV-1.22	5.71(5)	3.05(2)	534(6)	5.4(1)	1.69(3)	310(10)	5.0(2)	3.31(7)	660(30)
WS.HSV-1.24	140.05(9)	0.260(5)	1.86(3)	84.51(5)	0.270(4)	3.20(4)	74.80(5)	0.204(5)	2.73(6)

Supplementary Table 6. Quantified surface areas for the prefusion and postfusion conformations of diverse class III fusion machines.

Class III fusion machine	Prefusion PDB ID	Postfusion PDB ID	Missing residues in either conformation[#]	Prefusion surace area (Å^2)	Postfusion surace area (Å^2)	Prefusion solvent exposed area (%)	Postfusion solvent exposed area (%)
Baculovirus gp64	8YG6	3DUZ	272-278, 394-393, 461-487	56614.45	51037.45	4239.39 (7.49%)	10803.23 (21.17%)
HCMV gB	8VYN	8VYM	79-86, 115-119, 442-473, 699-700	129586.77	183572.46	10421.46 (8.04%)	20036.60 (10.91%)
HSV-1 gB	This study	This study	109-110, 174-184, 252-266, 460-500, 722-725	60503.22	64161.43	10010.67 (16.55%)	9107.83 (14.20%)
VSV G	5I2S	5I2M	411-413	47107.49	45736.07	4700.95 (9.98%)	5341.50 (11.68%)

Supplementary Table 7. Cryo-EM data collection, refinement and validation statistics for apo structures.

	**Apo gB-Ecto.516P** (EMDB-46765) (PDB 9DDC)	**Apo gB-Ecto.516P.531E** (EMDB-46763) (PDB 9DDB)	**Apo gB-Ecto.516P.531E.DS** (EMDB-46762) (PDB 9DDA)
**Data collection and processing**			
Magnification	105,000x	105,000x	105,000x
Voltage (kV)	300	300	300
Electron exposure (e–/Å^2)	58	58	58
Defocus range (μm)	-0.8 to -2.0	-0.8 to -2.0	-0.8 to -2.0
Pixel size (Å)	0.83	0.83	0.83
Symmetry imposed	C3	C3	C3
Initial particle images (no.)	1,297,859	2,212,583	3,110,069
Final particle images (no.)	78,265	169,306	223,040
Map resolution (Å)	2.87	3.71	2.99
FSC threshold	0.143	0.143	0.143
Map resolution range (Å)			
**Refinement**			
Initial model used (PDB code)	2GUM	6Z9M	6Z9M
Model resolution (Å)	2.8	3.7	3.0
FSC threshold	0.143	0.143	0.143
Model resolution range (Å)			
Map sharpening *B* factor (Å^2)	86.2	148.8	108.3
Model composition			
Non-hydrogen atoms	14,754	13,821	13,818
Protein residues	1,800	1,680	1,680
Ligands	15	21	21
B factors (Å^2)			
Protein	111.05	147.65	131.15
Ligand	140.96	153.39	167.67
R.m.s. deviations			
Bond lengths (Å)	0.005	0.004	0.005
Bond angles (°)	1.073	0.724	0.951
Validation			
MolProbity score	0.90	1.48	1.43
Clashscore	1.52	3.51	3.22
Poor rotamers (%)	0.00	0.00	0.00
Ramachandran plot			
Favored (%)	97.99	95.17	95.41
Allowed (%)	2.01	4.83	4.59
Disallowed (%)	0.00	0.00	0.00

Supplementary Table 8. Cryo-EM data collection, refinement and validation statistics for D48 complex structures.

	**D48 Fab in complex with gB-Ecto.516P** (EMDB-46760) (PDB 9DD8)	**D48 Fab in complex with gB-Ecto.516P.531E** (EMDB-46761) (PDB 9DD9)	**D48 Fab in complex with gB-Ecto.516P.531E.DS** (EMDB-467659) (PDB 9DD7)
**Data collection and processing**			
Magnification	105,000x	105,000x	105,000x
Voltage (kV)	300	300	300
Electron exposure (e–/Å^2)	58	58	58
Defocus range (μm)	-0.8 to -2.0	-0.8 to -2.0	-0.8 to -2.0
Pixel size (Å)	0.83	0.83	0.83
Symmetry imposed	C3	C3	C3
Initial particle images (no.)	2,044,718	1,090,332	1,351,706
Final particle images (no.)	139,193	43,574	73,388
Map resolution (Å)	3.08	3.77	4.14
FSC threshold	0.143	0.143	0.143
Map resolution range (Å)			
**Refinement**			
Initial model used (PDB code)	2GUM	2GUM	6Z9M
Model resolution (Å)	3.0	3.7	4.1
FSC threshold	0.143	0.143	0.143
Model resolution range (Å)			
Map sharpening *B* factor (Å^2)	78.6	118.3	155.3
Model composition			
Non-hydrogen atoms	19,854	19,710	17,502
Protein residues	2,439	2,418	2,175
Ligands	33	33	24
B factors (Å^2)			
Protein	97.70	98.17	153.16
Ligand	112.45	112.45	187.76
R.m.s. deviations			
Bond lengths (Å)	0.006	0.005	0.005
Bond angles (°)	1.187	0.892	1.193
Validation			
MolProbity score	1.26	1.25	1.57
Clashscore	3.13	3.00	3.29
Poor rotamers (%)	0.00	0.00	0.00
Ramachandran plot			
Favored (%)	97.14	97.11	92.92
Allowed (%)	2.86	4.89	7.08
Disallowed (%)	0.00	0.00	0.00

Supplementary Table 9. Cryo-EM data collection, refinement and validation statistics for WS.HSV-1.24 complex structure.

	**WS.HSV-1.24 Fab in complex with gB-Ecto.516P.531E** (EMDB-46758) (PDB 9DD6)
**Data collection and processing**	
Magnification	105,000x
Voltage (kV)	300
Electron exposure (e–/Å^2)	58
Defocus range (μm)	-0.8 to -2.0
Pixel size (Å)	0.83
Symmetry imposed	C3
Initial particle images (no.)	797,626
Final particle images (no.)	127,859
Map resolution (Å)	3.14
FSC threshold	0.143
Map resolution range (Å)	
**Refinement**	
Initial model used (PDB code)	6Z9M
Model resolution (Å)	3.1
FSC threshold	0.143
Model resolution range (Å)	
Map sharpening *B* factor (Å^2)	104.3
Model composition	
Non-hydrogen atoms	18,915
Protein residues	2,346
Ligands	21
B factors (Å^2)	
Protein	145.85
Ligand	184.36
R.m.s. deviations	
Bond lengths (Å)	0.005
Bond angles (°)	1.166
Validation	
MolProbity score	1.32
Clashscore	2.59
Poor rotamers (%)	0.00
Ramachandran plot	
Favored (%)	95.97
Allowed (%)	4.03
Disallowed (%)	0.00
