# Supplementary material for: Prefusion structure, evasion and neutralization of HSV-1 glycoprotein B
Source: Nat Microbiol. 2025 Oct 31;10(11):2966–80. doi: 10.1038/s41564-025-02153-x (PMC12578645; doi:10.1038/s41564-025-02153-x)
Supplement: Supplementary file 2 — Reporting Summary [file 41564_2025_2153_MOESM2_ESM.pdf]

nature portfolio

Corresponding author(s): Lawrence Shapiro; Peter D. Kwong

Last updated by author(s): Aug 18, 2025

Reporting Summary

Nature Portfolio wishes to improve the reproducibility of the work that we publish. This form provides structure for consistency and transparency in reporting. For further information on Nature Portfolio policies, see our Editorial Policies and the Editorial Policy Checklist.

Statistics

For all statistical analyses, confirm that the following items are present in the figure legend, table legend, main text, or Methods section.

n/a	Confirmed	
☐	☒	The exact sample size (n) for each experimental group/condition, given as a discrete number and unit of measurement
☒	☐	A statement on whether measurements were taken from distinct samples or whether the same sample was measured repeatedly
☐	☒	The statistical test(s) used AND whether they are one- or two-sided<br>*Only common tests should be described solely by name; describe more complex techniques in the Methods section.*
☒	☐	A description of all covariates tested
☒	☐	A description of any assumptions or corrections, such as tests of normality and adjustment for multiple comparisons
☐	☒	A full description of the statistical parameters including central tendency (e.g. means) or other basic estimates (e.g. regression coefficient) AND variation (e.g. standard deviation) or associated estimates of uncertainty (e.g. confidence intervals)
☒	☐	For null hypothesis testing, the test statistic (e.g. F, t, r) with confidence intervals, effect sizes, degrees of freedom and P value noted<br>*Give P values as exact values whenever suitable.*
☒	☐	For Bayesian analysis, information on the choice of priors and Markov chain Monte Carlo settings
☒	☐	For hierarchical and complex designs, identification of the appropriate level for tests and full reporting of outcomes
☒	☐	Estimates of effect sizes (e.g. Cohen's d, Pearson's r), indicating how they were calculated

Our web collection on statistics for biologists contains articles on many of the points above.

Software and code

Policy information about availability of computer code

Data collection	Leginon for automated cryo-EM data collection on FEI Titan Krios.
Data analysis	GraphPad Prism for data visualization and statistical analysis, including the visualization of means and standard deviations. CryoSPARC for cryo-EM data processing. GlycoSHIELD and GLYCO for modeling N-linked glycosylation. PDBePISA for solvent-accessibility analysis.

For manuscripts utilizing custom algorithms or software that are central to the research but not yet described in published literature, software must be made available to editors and reviewers. We strongly encourage code deposition in a community repository (e.g. GitHub). See the Nature Portfolio guidelines for submitting code & software for further information.

Data

Policy information about availability of data

All manuscripts must include a data availability statement. This statement should provide the following information, where applicable:

- Accession codes, unique identifiers, or web links for publicly available datasets
- A description of any restrictions on data availability
- For clinical datasets or third party data, please ensure that the statement adheres to our policy

There are no restrictions on data availability. The coordinates for the seven cryo-EM structures presented in this study are available under PDB accession codes 9DD6, 9DD7, 9DD8, 9DD9, 9DDA, 9DDB, and 9DDC. The corresponding cryo-EM 3D reconstruction maps for these coordinates are available under EMDB access codes 46758-46763 and 46765.

Research involving human participants, their data, or biological material

Policy information about studies with human participants or human data. See also policy information about sex, gender (identity/presentation), and sexual orientation and race, ethnicity and racism.

Reporting on sex and gender	n/a
Reporting on race, ethnicity, or other socially relevant groupings	n/a
Population characteristics	n/a
Recruitment	n/a
Ethics oversight	n/a

Note that full information on the approval of the study protocol must also be provided in the manuscript.

Field-specific reporting

Please select the one below that is the best fit for your research. If you are not sure, read the appropriate sections before making your selection.

☒ Life sciences ☐ Behavioural & social sciences ☐ Ecological, evolutionary & environmental sciences

For a reference copy of the document with all sections, see nature.com/documents/nr-reporting-summary-flat.pdf

Life sciences study design

All studies must disclose on these points even when the disclosure is negative.

Sample size	10 mice per immunization group will provide statistical power of 0.8 with alpha of 0.05 to confidently measure differences of at least 20% in polyclonal antibody neutralization titers elicited by each immunogen. This sample size of 10 per mice immunization group is the long-used standard in the field.
Data exclusions	No data was excluded.
Replication	All assays were performed in duplicate or triplicate to ensure reproducibility within experiments. All neutralization assays were performed twice independently to ensure reproducibility between experiments.
Randomization	Each immunization group received an even split of male and female mice to prevent any bias from sex. All mice were the same age when purchased, so this was not a factor that needed to be randomized. No other randomization or covariation applied to this study.
Blinding	Investigators performing ELISAs and virus neutralization assays with mouse sera were blinded sample immunization group so they did not know which immunogen the animal had been vaccinated with.

Reporting for specific materials, systems and methods

We require information from authors about some types of materials, experimental systems and methods used in many studies. Here, indicate whether each material, system or method listed is relevant to your study. If you are not sure if a list item applies to your research, read the appropriate section before selecting a response.

Materials & experimental systems

n/a	Involved in the study	
☐	☒	Antibodies
☐	☒	Eukaryotic cell lines
☒	☐	Palaeontology and archaeology
☐	☒	Animals and other organisms
☒	☐	Clinical data
☒	☐	Dual use research of concern
☒	☐	Plants

Methods

n/a	Involved in the study	
☒	☐	ChIP-seq
☐	☒	Flow cytometry
☒	☐	MRI-based neuroimaging

Antibodies

Antibodies used	All monoclonal antibodies used in this study were recombinantly expressed by our labs or obtained directly from other academic labs, none of which are commercially available. Antibodies D48 and hu2c had been previously published; genes encoding these antibodies were synthesized and cloned (GenScript) for recombinant expression. Antibodies DL16, SS10, and SS55 had been previously published; these antibodies were a gift from Gary Cohen at the University of Pennsylvania. Antibodies WS.HSV-1.02 through WS.HSV-1.24 are described in this study; genes encoding these antibodies were synthesized and cloned (GenScript) for recombinant expression. Polyclonal antibodies used for detecting mouse and human antibodies by ELISA were all purchased from Jackson Immuno: HRP-goat anti-mouse IgG (#115-035-008) or HRP-goat anti-human IgG (#109-035-088).
Validation	When available, binding affinities (as measured by ELISA and/or SPR) and neutralization IC50 titer (as measured by authentic HSV-1 neutralization) were validated against previously published data.

Eukaryotic cell lines

Policy information about cell lines and Sex and Gender in Research

Cell line source(s)	Expi293 cells were obtained from Thermo Fisher (#A14528). Vero cells were obtained from ATCC (#CCL-81).
Authentication	All cell lines were obtained directly from their respective vendor and therefore not authenticated.
Mycoplasma contamination	All cell lines are negative for mycoplasma contamination.
Commonly misidentified lines (See ICLAC register)	n/a

Animals and other research organisms

Policy information about studies involving animals; ARRIVE guidelines recommended for reporting animal research, and Sex and Gender in Research

Laboratory animals	C57BL/6J (Strain #:000664) and BALB/cJ (Strain #:000651) mice were purchased from The Jackson Laboratory. All mice were obtained at four weeks of age and began use in our studies at approximately six weeks of age.
Wild animals	The study did not involve wild animals.
Reporting on sex	Sex was considered in study design to ensure that each mouse immunization group contained an even amount of each sex (5 female and 5 male). Sex-based analyses were not specifically performed since this study was not designed to do so and there is no precedent for sex-based differences in antibody responses to viral protein immunizations in these mice strains.
Field-collected samples	The study did not involve samples collected from the field.
Ethics oversight	All mouse immunization experiments were reviewed and approved by the respective Institutional Animal Care and Use Committees of Columbia University and the Vaccine Research Center, NIAID, NIH.

Note that full information on the approval of the study protocol must also be provided in the manuscript.

Plants

Seed stocks	n/a
Novel plant genotypes	n/a
Authentication	n/a

Flow Cytometry

Plots

Confirm that:

☒ The axis labels state the marker and fluorochrome used (e.g. CD4-FITC).

☒ The axis scales are clearly visible. Include numbers along axes only for bottom left plot of group (a 'group' is an analysis of identical markers).

☒ All plots are contour plots with outliers or pseudocolor plots.

☐ A numerical value for number of cells or percentage (with statistics) is provided.

Methodology

Sample preparation	Flow cytometry analysis was used to assess antibody recognition of full-length HSV-1 gB expressed on the surface of Expi293 cells (Thermo Fisher #A14528) by transient transfection. Cells were analyzed after 40 hours of expression of HSV-1 gB from transfected plasmid under the control of a CMV promoter. No tissues or blood samples or other types of biological samples obtained from animal models or human donors were used, and no specific cell populations were defined except for those based on viability.
Instrument	BD Fortessa
Software	FlowJo version 10.10.0
Cell population abundance	n/a
Gating strategy	Gating was used to simply select live cells for analysis, since the transiently transfected HSV-1 gB construct does not have a fluorescent tag to report expression. FSC/SSC was used to select the Expi293 cells, an immortalized mammalian cell line. FSC-H and FSC-A was used to gate on singlets. LiveDead Violet was used to gate on live cells. An example of the gating strategy is provided as a Supplementary Figure.

☒ Tick this box to confirm that a figure exemplifying the gating strategy is provided in the Supplementary Information.
